# LAWS-HiC: A Locally Adaptive Weighting and Screening (LAWS) Approach to Improve Detection of Long-Range Chromatin Interactions from Hi-C Data

**DOI:** 10.3390/mps9050135

**Published:** 2026-09-18

**Authors:** Lingbo Zhou, Chang Chen, Jane Zizhen Zhao, Ming Hu, Yun Li

**Affiliations:** 1 Department of Biostatistics, University of North Carolina at Chapel Hill, 135 Dauer Drive, Chapel Hill, NC 27599, USA; lzhou1@unc.edu (L.Z.);; 2 Department of Psychology and Neuroscience, University of North Carolina at Chapel Hill, 235 East Cameron Avenue, Chapel Hill, NC 27599, USA; jan3zhao@unc.edu; 3 Department of Genomic Sciences and Systems Biology, Cleveland Clinic Research, Cleveland Clinic Foundation, 9500 Euclid Avenue, Cleveland, OH 44195, USA; 4 Department of Genetics, University of North Carolina at Chapel Hill, 120 Mason Farm Road, Chapel Hill, NC 27599, USA; 5 Department of Computer Science, University of North Carolina at Chapel Hill, 201 S Columbia Street, Chapel Hill, NC 27599, USA

**Keywords:** Hi-C, chromatin interactions, peak calling, spatial multiple testing, false discovery rate, topologically associating domains, 3D genome organization

## Abstract

Hi-C technologies are widely used to study genome-wide chromosome spatial organization. Among various Hi-C data analyses, detecting long-range chromatin interactions (i.e., 3D peak calling) is particularly critical due to its direct relevance to gene regulation. However, most existing peak callers fail to account for spatial dependencies in high-resolution (e.g., ≤10 kb) Hi-C data, often resulting in suboptimal accuracy. To address this limitation, we introduce LAWS-HiC, a novel computational method based on the Locally Adaptive Weighting and Screening (LAWS) approach. LAWS-HiC adjusts *p*-values from a standard Hi-C peak caller by incorporating local spatial dependence within topologically associating domains (TADs) in a data-driven manner. Benchmarking in two deeply sequenced Hi-C datasets from human lymphoblastoid cell line GM12878 and mouse embryonic stem cells (mESCs), LAWS-HiC consistently improves the area under the precision–recall curve (PRAUC) across varying sequencing depths, with larger gains at lower sequencing depths. At the standard Benjamini–Hochberg false discovery rate (BH-FDR) ≤ 0.05, biological feature overlap analysis confirms that LAWS-HiC calls were more strongly enriched for regulatory annotations relative to non-calls than those from alternative methods. LAWS-HiC is freely available as an R package on GitHub.

## 1. Introduction

Three-dimensional (3D) organization of chromatin plays a key role in genome structure and genome function [1,2]. Since first invented in 2009, Hi-C [3] and its derived technologies (e.g., Micro-C [4], ChIA-PET [5], PLAC-seq [6], HiChIP [7], and in situ DNase Hi-C [8]) have been widely used to map genome-wide chromosome conformation. The bulk Hi-C data, capturing genome-wide chromatin interaction frequency in a population of cells, can be summarized into a contact frequency matrix. Alongside the rapid improvement of sequencing technology, computational tools are needed to analyze and interpret Hi-C data. Among various Hi-C data analyses, the detection of significant chromatin interactions (i.e., 3D peak calling) is particularly critical, as long-range chromatin loops can bring *cis*-regulatory elements, such as distal enhancers, into close spatial proximity with their target gene’s promoter to regulate gene expression. Statistically, 3D peak calling involves testing against the null hypothesis that chromatin contact frequencies are solely the result of random chromatin collisions. Genomic loci pairs with higher-than-expected contact frequencies are defined as 3D peaks.

Several computational tools, such as HiCCUPS [9], Fit-Hi-C [10], FitHiC2 [11], HiC-DC [12] and Mustache [13], have been proposed to detect 3D peaks from Hi-C data. For instance, HiCCUPS [9] identifies peaks by detecting enriched bin pairs in the contact matrix whose contact frequencies significantly exceed those of surrounding loci in multiple directions, using a fixed-size local neighborhood. In contrast, other tools rely on global background models. Fit-Hi-C [10] models a null contact probability using a distance-dependent monotonic spline and a coverage-based correction, then estimates the statistical significance of each interaction using a binomial distribution; FitHiC2 [11], a reimplementation of Fit-Hi-C, extends this framework to efficiently perform genome-wide analysis for high-resolution Hi-C data; similarly, HiC-DC [12] employs a zero-truncated negative binomial regression to account for zero inflation, over-dispersion, and other systematic biases such as genomic distance. Beyond these dedicated peak callers, other tools improve the detectability of 3D peaks by refining the Hi-C signal upstream: HiCorr [14] applies a rigorous bias-correction pipeline that increases the sensitivity and robustness of loop identification, particularly for enhancer–promoter interactions, while HiCNet [15] reconstructs high-resolution three-dimensional chromosomal structures from which TADs and DNA loops indicative of enhancer–promoter interactions can be recovered. However, most of these methods assume that chromatin contact frequencies between pairs of genomic loci are statistically independent. This assumption is often invalid, especially for high-resolution Hi-C data (e.g., kilobase resolution), where neighboring pairs exhibit strong correlation. The inaccurate independence assumption can result in reduced sensitivity and an inflated false positive rate. A promising solution is to post-process the results of state-of-the-art peak callers using methods that account for spatial dependencies. For example, our group recently developed HiC-ACT [16], a summary statistics-based method that adjusts *p*-values from standard peak callers using the aggregated Cauchy test (ACT).

HiC-ACT accounts for spatial dependence by aggregating the *p*-values of neighboring bin pairs into a combined test statistic so that the significance assigned to a bin pair reflects the evidence pooled across its neighborhood. An alternative is to use the neighborhood to estimate how sparsely true interactions are distributed around each bin pair and to use that estimate to adjust the bin pair’s own *p*-value accordingly. Here we present LAWS-HiC, a computational method that leverages the Locally Adaptive Weighting and Screening (LAWS) approach [17] to adjust for correlations between nearby loci pairs. LAWS-HiC incorporates spatial information by adaptively estimating the local sparsity level of data, using the position identifiers of loci pairs and corresponding *p*-values returned by an upstream peak caller. LAWS-HiC is therefore applicable to peak callers that report a *p*-value for each bin pair included in the analysis. To account for the uncertainty in single-threshold sparsity estimation, LAWS-HiC aggregates sparsity estimates over an ensemble of screening thresholds within each topologically associating domain (TAD). Moreover, LAWS-HiC is fully data-driven and does not require any prior knowledge or assumptions about the spatial pattern of the original Hi-C data.

## 2. Materials and Methods

### 2.1. Hi-C Datasets and Down-Sampling

We applied LAWS-HiC to two deeply sequenced Hi-C datasets. The Hi-C data from the GM12878 human lymphoblastoid cell line was obtained from Rao et al. [9], aggregated at 10 kb resolution and comprising approximately 4.9 billion intra-chromosomal contact reads across the autosomes, with TAD boundaries taken from the original publication. The Hi-C data from mouse embryonic stem cells (mESCs) was obtained from Bonev et al. [18], aggregated at 10 kb resolution and comprising approximately 7.2 billion intra-chromosomal contact reads across the autosomes, with TAD boundaries called using the insulation-score algorithm of Crane et al. [19]. For both datasets, the input *p*-values supplied to LAWS-HiC were obtained by running FitHiC2 [11] on the corresponding contact maps.

To evaluate LAWS-HiC across realistic sequencing depths, we generated down-sampled versions of each dataset by multinomial thinning of the observed contact counts, corresponding to the “Random - no weight” strategy described by Oliveira Junior et al. [20]. For each chromosome, the down-sampled contact counts were drawn from a multinomial distribution with total count equal to r×N, where *r* is the target down-sampling fraction and *N* is the chromosome’s total read count, with sampling probabilities proportional to the original contact counts. Down-sampling was performed independently for each chromosome, without additional weighting by genomic distance or contact frequency. For both GM12878 and mESC, we generated datasets targeting total intra-chromosomal read counts of 0.25, 0.50, 0.75, and 1.00 billion, corresponding to 5%, 10%, 15%, and 20% of the original depth for GM12878 and 4%, 7%, 11%, and 14% for mESC. For each sequencing depth, the down-sampled dataset used for method comparisons represented a single random realization generated without a fixed random seed. FitHiC2 was then run on each down-sampled contact matrix to produce the *p*-values used as input to LAWS-HiC and HiC-ACT.

To assess variability due to random down-sampling, we generated five independent replicates at the shallowest sequencing depth (0.25 billion intra-chromosomal reads) for each cell line and repeated the precision–recall evaluation for each replicate. Random seeds were 20260801-20260805 for GM12878 and 20260811-20260815 for mESC.

### 2.2. LAWS-HiC Algorithm

The Locally Adaptive Weighting and Screening (LAWS) framework [17] is a spatial multiple testing procedure in which each *p*-value is reweighted according to the estimated local sparsity of its surrounding neighborhood. At each location in a spatial grid, LAWS estimates a local sparsity level, which is the probability that the location harbors a true signal, by spatially smoothing the empirical proportion of locations whose *p*-values exceed a threshold τ. The estimated sparsity is then converted into a location-specific adaptive weight, and each *p*-value is divided by its weight: locations in signal-dense regions receive smaller adjusted *p*-values, while locations in sparse regions receive larger ones. We adapt this framework to two-dimensional Hi-C contact matrices as follows. Formal derivations appear in Supplementary Methods.

Let p(ij) denote the initial *p*-value for a candidate long-range chromatin interaction between bins *i* and *j*, obtained from FitHiC2. The LAWS-adjusted *p*-value is

(1) $$p^{\mathrm{laws}}\left(ij\right)=\min\left\{\frac{p(ij)}{\hat{w}\left(ij\right)},\;1\right\},\;\hat{w}\left(ij\right)=\frac{\hat{\pi }\left(ij\right)}{1-\hat{\pi }\left(ij\right)},$$

where $\hat{\pi }\left(ij\right)$ is an estimate of the local sparsity level at bin pair (i,j).

Local sparsity estimation within TADs.

Because including all bin pairs across a chromosome is computationally intractable, we restrict the neighborhood set S for spatial pooling to bin pairs located within the same topologically associating domain (TAD). For each pair (i,j)∈S, a Gaussian kernel assigns smoothing weights to neighboring pairs (m,n)∈S:

(2) $$v\left(ij,mn\right)=\exp\;\left(-\frac{{\left(i-m\right)}^{2}+{\left(j-n\right)}^{2}}{2h^{2}}\right),$$

where *h* is the smoothing bandwidth. Following Cai et al. [17], the local sparsity estimator at threshold τ takes the form

(3) $${\hat{\pi }}_{\tau }\left(ij\right)=1-\frac{\sum_{\left(m,n)\in T(\tau \right)}v\left(ij,mn\right)}{\left(1-\tau \right)\sum_{(m,n)\in S}v\left(ij,mn\right)},$$

where T(τ)={(i,j)∈S:p(ij)>τ} is the screening subset of bin pairs treated as candidate nulls.

Multi-threshold ensemble.

Rather than committing to a single threshold τ, which imposes a fragile binary partition between candidate-null and candidate-signal bin pairs (Supplementary Methods), LAWS-HiC aggregates ${\hat{\pi }}_{\tau }\left(ij\right)$ over a grid of K=10 thresholds. Within each TAD, the thresholds are placed at evenly spaced quantiles of the empirical *p*-value distribution between the 20th and 95th percentiles. The final sparsity estimate is the weighted average

(4) $$\hat{\pi }\left(ij\right)=\sum_{k=1}^{K}\omega_{k}\;{\hat{\pi }}_{\tau_{k}}\left(ij\right),\;\omega_{k}\propto \sqrt{\left|T(\tau_{k})\right|},\;\sum_{k=1}^{K}\omega_{k}=1.$$

The weighting rule $\omega_{k}\propto \sqrt{\left|T(\tau_{k})\right|}$ is proportional to the inverse standard error of ${\hat{\pi }}_{\tau_{k}}\left(ij\right)$ and reflects a deliberate bias–variance trade-off detailed in Supplementary Methods. Each per-threshold estimate ${\hat{\pi }}_{\tau_{k}}\left(ij\right)$ is clipped to $\left[10^{-5},\;1-10^{-5}\right]$ for numerical stability.

Bandwidth selection and small-TAD handling.

The bandwidth *h* is set adaptively as h=max(2,αL), where *L* is the TAD length in bins and α=0.10. This integrates information over at least 10% of the TAD’s genomic span, with a minimum of 2 bins. TADs shorter than 100 kb or containing fewer than 20 bin pairs in S are excluded from LAWS-HiC adjustment; bin pairs within such TADs retain their original FitHiC2 *p*-values and are flagged in the output. Full justification of these choices appears in Supplementary Methods.

After the LAWS-HiC adjustment, the Benjamini–Hochberg (BH) procedure [21] is applied to the resulting *p*-value vector to produce false discovery rate (FDR)-adjusted *p*-values. This vector includes LAWS-adjusted *p*-values for eligible bin pairs and the original FitHiC2 *p*-values retained for bin pairs within the later excluded small TADs.

Sensitivity analyses for the bandwidth proportion α, the number of screening thresholds *K*, and the endpoints of the threshold quantile grid are reported in Supplementary Note S1. Performance was robust to the threshold-grid endpoints, with opposite preferences for the lower endpoint across the two cell lines, while the multi-threshold ensemble provided consistently near-optimal performance; PRAUC decreased monotonically with increasing α, indicating that the value used in the main analysis was conservative with respect to the bandwidth choice.

Relationship to HiC-ACT.

LAWS-HiC and HiC-ACT [16] both operate on the per-bin-pair *p*-values produced by a standard Hi-C peak caller, exploiting the spatial arrangement of those *p*-values over bin pairs through a Gaussian kernel defined on a local neighborhood, and both retain the *p*-value of the bin pair under test. The two methods differ, however, in how the neighborhood enters the inference. HiC-ACT combines the *p*-values of all bin pairs in the neighborhood into a single test statistic via the aggregated Cauchy test so that the magnitudes of neighboring *p*-values act directly on the significance assigned to the bin pair; as a consequence, the smoothed *p*-value is driven by small *p*-values in the neighborhood.

LAWS-HiC instead places the problem in the framework of simultaneous inference with auxiliary information [17], in which the spatial location serves as an auxiliary variable supplying structural information. Here the neighborhood is used only to estimate the local sparsity level $\hat{\pi }\left(ij\right)$, the proportion of bin pairs in the neighborhood that represent true interactions, through a screening step that counts how many neighboring *p*-values exceed a threshold τ. Because neighbors contribute to $\hat{\pi }\left(ij\right)$ as counts rather than magnitudes, no single extreme neighbor can dominate the estimate. The resulting weight $\hat{w}\left(ij\right)=\hat{\pi }\left(ij\right)/\left\{1-\hat{\pi }\left(ij\right)\right\}$ rescales the bin pair’s own *p*-value, up-weighting *p*-values in neighborhoods where interactions are abundant and down-weighting those where interactions are sparse. LAWS-HiC therefore adapts the stringency of the test to the estimated local signal density, whereas HiC-ACT adapts the evidence itself.

The empirical performance of LAWS-HiC is evaluated in Section 3 through benchmarking against HiC-ACT and FitHiC2 across two cell lines and multiple sequencing depths.

### 2.3. Competing Methods

We compared LAWS-HiC against two existing methods. FitHiC2 [11] (version 2.0.7; La Jolla Institute for Immunology, La Jolla, CA, USA) served as the baseline, and its *p*-values were used as input to both LAWS-HiC and HiC-ACT. FitHiC2 was run on the full-depth data and on each down-sampled dataset at 10 kb resolution for interactions spanning 20 kb to 2 Mb, with the default two spline-fitting passes. No bias file was supplied; consequently, all bin-level bias terms were set to unity and the fitted expectation depended only on genomic distance. HiC-ACT [16] (version 0.1.0; University of North Carolina at Chapel Hill, Chapel Hill, NC, USA) is a post-processing method that adjusts FitHiC2 *p*-values using the aggregated Cauchy test over a local neighborhood. We applied HiC-ACT with a smoothing bandwidth of 20 bins, as used in the original study, and an initial *p*-value threshold of $10^{-3}$. Bin pairs with FitHiC2 $p>10^{-3}$ retained their original FitHiC2 *p*-values.

Second upstream peak caller.

LAWS-HiC operates on per-bin-pair *p*-values provided by an upstream peak caller and is not specific to FitHiC2. To evaluate its applicability to a different upstream caller, we repeated the analysis using HiC-DC+ [22] in place of FitHiC2. HiC-DC+ models contact counts using negative binomial regression and provides a *p*-value for each bin pair, thereby satisfying the input requirements of LAWS-HiC. We used HiC-DC+ (implemented in HiCDCPlus version 1.14.0, Bioconductor 3.20; Memorial Sloan Kettering Cancer Center, New York, NY, USA) under R 4.4.0 at 10 kb resolution and restricted the analysis to the same 20 kb to 2 Mb genomic distance range used for FitHiC2. HiC-DC+ was applied to the same down-sampled contact matrices used as input to FitHiC2. The bin grid and tested bin pairs were derived directly from the same down-sampled contact matrices used for FitHiC2, ensuring identical input bin pairs for the two upstream callers. We fitted the distance-only HiC-DC+ model without GC content, mappability, or effective-length covariates because FitHiC2 was run without a bias file. Including these covariates only for HiC-DC+ would introduce an additional difference between the upstream callers beyond their statistical models. LAWS-HiC was then applied to the resulting HiC-DC+ *p*-values using the same parameter settings as in the FitHiC2-based analysis. The candidate set and truth-peak definitions were held fixed, as described below, so that the upstream *p*-value vector was the only input to the LAWS-HiC post-processing step that differed between the two analyses.

### 2.4. Evaluation Framework

For each dataset and sequencing depth, we defined the candidate set as all bin pairs within TAD regions satisfying (i) contact count in the original data > 15 and (ii) contact count in the down-sampled data > 5, expected count >5r (where *r* is the down-sampling fraction), and (iii) observed-to-expected ratio > 1.5. Both precision–recall analysis and biological evaluation were restricted to the candidate set. Following Lagler et al. [16], the truth peaks were defined as bin pairs with FitHiC2 *p*-value $<10^{-12}$ in the original data.

Precision-recall analysis.

For each combination of cell line, sequencing depth, and method, we computed precision–recall curves using the PRROC R package [23] (version 1.4; Martin Luther University Halle-Wittenberg, Institute of Computer Science, Halle (Saale), Germany) on bin pairs pooled across the autosomes and report the resulting area under the precision–recall curve (PRAUC). For statistical comparison across methods, we additionally computed per-chromosome PRAUC and summarized the paired chromosome-level differences using Hodges–Lehmann (HL) estimates and 95% confidence intervals [24]. We also recorded the number of autosomes on which LAWS-HiC achieved a higher PRAUC than each competing method.

Prevalence-rescaled PRAUC.

The PRAUC of a random ranking equals the prevalence ρ of truth peaks within the candidate set, so PRAUC values obtained under different prevalences are not directly comparable. We therefore also report prevalence-rescaled PRAUC, defined as (PRAUC−ρ)/(1−ρ), such that a value of 0 corresponds to random ranking and a value of 1 corresponds to perfect ranking, regardless of prevalence.

Orthogonal truth sets.

The primary truth set defined above was derived from full-depth FitHiC2 *p*-values, while FitHiC2 also served as the baseline and provided the input *p*-values for LAWS-HiC and HiC-ACT. To provide an evaluation independent of this FitHiC2-based truth definition, we repeated the precision–recall analysis using loop annotations derived from independent experimental data. For GM12878, we used the CTCF and RNA polymerase II (RNAPII) ChIA-PET loop sets of Tang et al. [25], evaluated separately and as their union. For mESC, we used Micro-C data from Hsieh et al. [26], with loops called independently by Mustache [13] and Chromosight [27], and evaluated the two loop sets separately. To allow a one-bin positional tolerance, each annotated loop anchor was extended by 10 kb on both sides before matching. A candidate bin pair was classified as a truth peak when its two 10 kb anchor bins overlapped the two extended loop anchors. For each sequencing depth, evaluation was restricted to the corresponding candidate set.

### 2.5. Biological Feature Overlap Evaluation

To evaluate differences in biological feature overlap among the methods, we defined the call set for each method (LAWS-HiC, HiC-ACT, and FitHiC2) as bin pairs called at BH-FDR ≤ 0.05. The not-called set consisted of all remaining bin pairs in the candidate set. As a complementary comparison, we also defined a rejected set for each method as bin pairs not called by that method but called by at least one of the other two methods.

Biological features and overlap definitions.

For GM12878, we used CTCF and RNAPII ChIA-PET loop data from Tang et al. [25] and enhancer annotations from the Roadmap Epigenomics Consortium [28], including 10,335 typical enhancers and 252 super enhancers. A bin pair was defined to overlap a ChIA-PET interaction when its two anchors jointly overlapped the two anchors of the ChIA-PET interaction. For the Roadmap super-enhancer and typical-enhancer annotations, a bin pair was defined to overlap the feature when either of its two anchors overlapped an annotated enhancer region. An enhancer–promoter (E-P) interaction in GM12878 was defined as a bin pair in which one anchor overlapped an enhancer annotation and the other anchor overlapped the promoter region of an expressed gene [29], with the promoter defined as ±500 bp around the transcription start site (TSS) [30]. For mESC, we used ChIP-seq peaks for CTCF, H3K4me1, H3K4me3, H3K27ac [31,32,33], ATAC-seq peaks [33], super-enhancer annotations from dbSUPER [34], and active enhancer annotations from FANTOM5 [35]. For these individual 1D features, a bin pair was defined to overlap the feature when either of its two anchors overlapped the feature’s genomic location. An E-P interaction in mESC was defined as a bin pair in which one anchor overlapped the dbSUPER or FANTOM5 enhancer annotation, and the other anchor overlapped the TSS [30] ±500 bp of an mESC-expressed gene [36] (TPM > 1 or FPKM > 1).

Statistical tests.

For each method and biological feature, we compared the call set with the not-called set and, separately, with the rejected set. For each 2×2 table, we report the odds ratio (OR) and its 95% confidence interval. When all four cell counts were at least 5, the confidence interval was calculated on the log-OR scale using the Woolf method [37], with a chi-square test without continuity correction. When any cell count was below 5, we used an exact conditional confidence interval and Fisher’s exact test. All call-vs.-not-call comparisons used the Woolf interval and chi-square test; the exact procedure was required only for some call-vs.-rejected comparisons.

## 3. Results

### 3.1. LAWS-HiC Consistently Improves PRAUC Across Cell Types and Sequencing Depths

We first evaluated the overall accuracy of LAWS-HiC, HiC-ACT, and FitHiC2 using the area under the precision–recall curve (PRAUC), computed per chromosome and aggregated across the autosomes for each combination of cell type and sequencing depth. Table 1 reports the autosome-pooled PRAUC for each method across all eight conditions, together with paired comparison between LAWS-HiC and each competing method across chromosomes.

For GM12878 Hi-C data, LAWS-HiC achieved autosome-pooled raw PRAUCs of 0.952, 0.941, 0.945, and 0.950 at 0.25, 0.50, 0.75, and 1.00 billion reads, respectively, compared to 0.939, 0.930, 0.930, and 0.924 for HiC-ACT and 0.935, 0.929, 0.937, and 0.945 for FitHiC2. For mESC Hi-C data, LAWS-HiC achieved PRAUCs of 0.925, 0.886, 0.876, and 0.878 at the four depths, compared to 0.912, 0.874, 0.866, and 0.865 for HiC-ACT and 0.903, 0.862, 0.856, and 0.863 for FitHiC2. The apparent non-monotonic pattern in raw PRAUC across sequencing depths primarily reflected the decline in truth-set prevalence, from 0.813 to 0.637 in GM12878 and from 0.800 to 0.465 in mESC, as shown in Table 1, which changes the random-ranking baseline of the precision–recall curve. After rescaling PRAUC to a common baseline, PRAUC increased monotonically with sequencing depth for all three methods in both cell lines. LAWS-HiC retained the highest prevalence-rescaled PRAUC in all eight conditions. Across all eight combinations of cell line and sequencing depth, LAWS-HiC achieved higher per-chromosome PRAUC than both HiC-ACT and FitHiC2 on every autosome (22/22 in GM12878 and 19/19 in mESC). The Hodges–Lehmann estimates [24], defined as the median of all pairwise differences between paired chromosome-level observations, ranged from +0.009 to +0.027 for the per-chromosome PRAUC differences between LAWS-HiC and HiC-ACT and from +0.005 to +0.023 for those between LAWS-HiC and FitHiC2.

Across five independent down-sampling replicates at 0.25 billion reads, the standard deviation of the paired PRAUC difference between LAWS-HiC and each competing method was below 0.001 in both cell lines, whereas the corresponding mean differences ranged from 0.013 to 0.022. LAWS-HiC also achieved the highest PRAUC on every autosome in all five replicates for both cell lines (Table S1).

The truth set used above was defined from full-depth FitHiC2 *p*-values, while FitHiC2 also served as the upstream caller. We therefore repeated the precision–recall analysis using five orthogonal loop annotation sets: the CTCF and RNAPII ChIA-PET loop sets and their union in GM12878 [25], and Micro-C loops called by Mustache and Chromosight in mESC [26] (Table S2). Absolute performance was substantially lower under these orthogonal truth sets. For LAWS-HiC, prevalence-rescaled PRAUC ranged from 0.210 to 0.358 in GM12878 and from 0.218 to 0.259 in mESC, compared with 0.741–0.863 and 0.624–0.772, respectively, under the FitHiC2-based truth set. LAWS-HiC nevertheless achieved higher prevalence-rescaled PRAUC than FitHiC2 in all 20 combinations of orthogonal truth set and sequencing depth, with differences ranging from 0.002 to 0.079. The comparison with HiC-ACT depended more strongly on the choice of truth set. LAWS-HiC was higher in 10 of the 20 conditions overall: at all four depths for the CTCF truth set, none of the four depths for RNAPII, two of four depths for the ChIA-PET union, three of four depths for Mustache, and one of four depths for Chromosight.

To assess whether the improvement provided by LAWS-HiC depends specifically on FitHiC2 as the upstream caller, we repeated the evaluation using HiC-DC+ [22] (Table S3). LAWS-HiC also increased prevalence-rescaled PRAUC when applied to HiC-DC+ *p*-values. Under the FitHiC2-based truth set, the gain over HiC-DC+ in mESC was 0.225, 0.171, 0.131, and 0.099 across the four sequencing depths, compared with gains of 0.108, 0.062, 0.041, and 0.028 over FitHiC2. In GM12878, the corresponding gains over HiC-DC+ were 0.139 and 0.051 at the two shallower depths, compared with 0.088 and 0.041 over FitHiC2. The same pattern was observed using the orthogonal truth sets selected for this comparison. With Mustache loops in mESC, the gain over HiC-DC+ was 0.095, 0.094, 0.080, and 0.065 and exceeded the corresponding gain over FitHiC2 at every depth. With the ChIA-PET union in GM12878, the gain over HiC-DC+ was 0.052 and 0.018 at the two shallower depths. Under both truth-set definitions used in this analysis, the gain, defined as the difference in prevalence-rescaled PRAUC between LAWS-HiC and the corresponding upstream caller, decreased monotonically with increasing sequencing depth in both cell lines (Figure S1). The decline was steeper for HiC-DC+ in GM12878, where the gain became slightly negative at the two deepest depths under the FitHiC2-based truth set (−0.002 and −0.038) and at the deepest depth under the ChIA-PET union (−0.009).

### 3.2. LAWS-HiC Achieves Higher Precision at Fixed FDR Thresholds and Matched Call-Set Sizes

**Figure 1:**
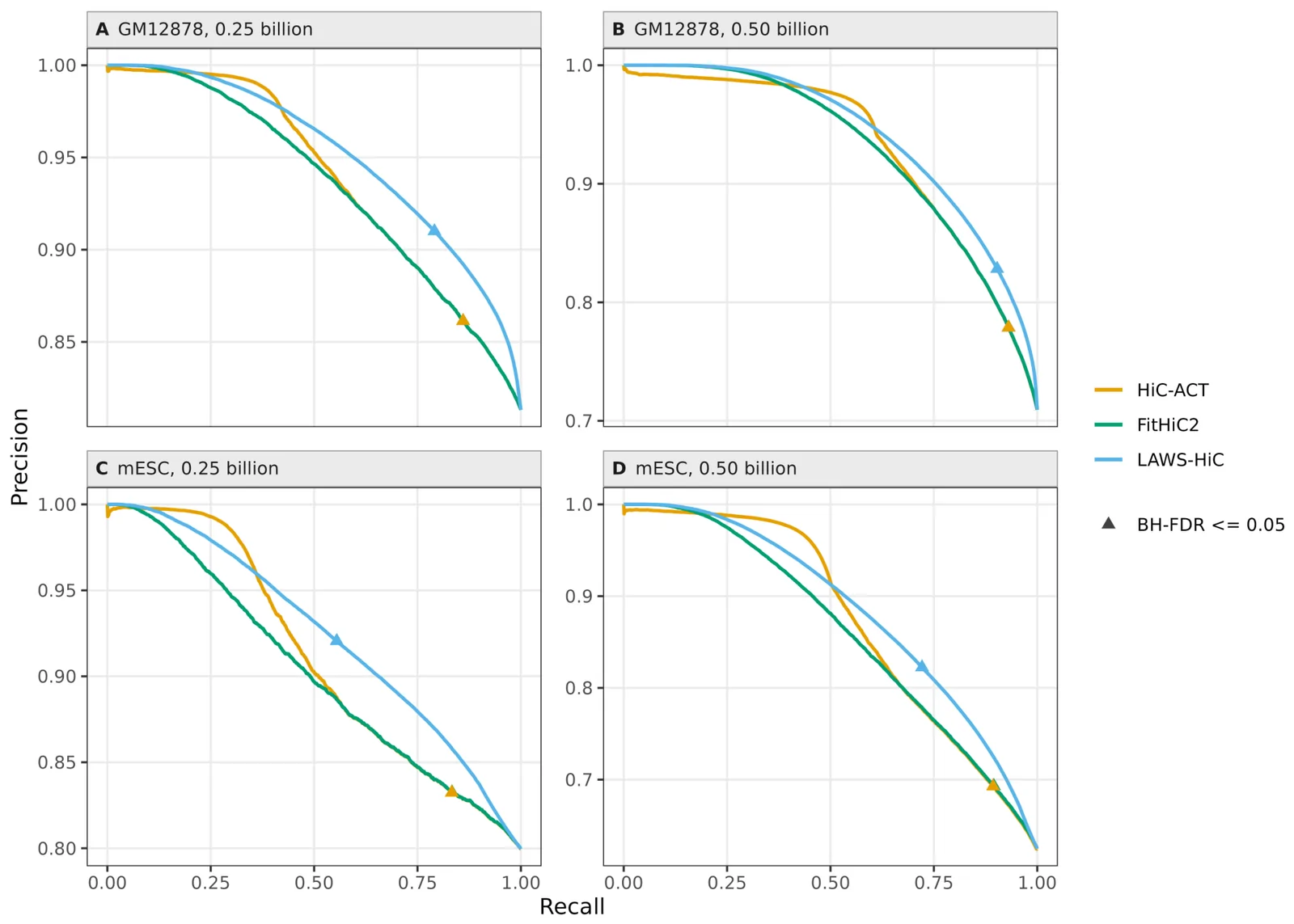
Precision–recall curves of LAWS-HiC (blue), HiC-ACT (orange), and FitHiC2 (green) at the 0.25 billion and 0.50 billion sequencing depths for GM12878 and mESC cells. (**A**) GM12878 at 0.25 billion reads. (**B**) GM12878 at 0.50 billion reads. (**C**) mESC at 0.25 billion reads. (**D**) mESC at 0.50 billion reads. The truth set used to compute precision and recall is defined as bin pairs in the candidate set with full-depth FitHiC2 *p*-value $<10^{-12}$ (Section 2.4). Triangles mark each method’s operating point at Benjamini–Hochberg false discovery rate (BH-FDR) ≤ 0.05. Precision–recall curves for the deeper sequencing depths (0.75 and 1.00 billion reads) are shown in Figures S2 and S3.

To examine the precision–recall performance underlying the PRAUC differences, we compared the full precision–recall curves across the two cell lines and four sequencing depths. Figure 1 shows the curves at the two shallowest depths (0.25 and 0.50 billion reads) for both cell lines, with the corresponding results at 0.75 and 1.00 billion reads shown in Figures S2 and S3. Across the eight conditions, the relative ordering of the three curves changed across the recall range and with sequencing depth. LAWS-HiC showed higher precision over substantial portions of the precision–recall range, with the clearest separation from the competing methods observed at the shallower sequencing depths.

We next compared precision and recall at fixed BH-FDR thresholds of 0.01, 0.05, and 0.10 (Table 2). LAWS-HiC achieved higher precision than both HiC-ACT and FitHiC2 in all 24 combinations of sequencing depth and FDR threshold. At BH-FDR ≤ 0.05, the precision gain over FitHiC2 ranged from 0.038 to 0.050 in GM12878 and from 0.088 to 0.136 in mESC, with similar improvements observed at BH-FDR thresholds of 0.01 and 0.10. At the same nominal FDR, the higher precision of LAWS-HiC was accompanied by lower recall, consistent with its smaller call set. At a nominal BH-FDR ≤ 0.05, the number of bin pairs called by LAWS-HiC was 87–94% of that called by FitHiC2 in GM12878 and 60–75% in mESC.

With empirical FDR defined as 1−precision, at a nominal BH-FDR of 0.05, it ranged from 0.079 to 0.332 for LAWS-HiC and from 0.139 to 0.459 for HiC-ACT and FitHiC2, exceeding the nominal level for all three methods in all eight conditions (Table 2). The same pattern was observed at nominal BH-FDR thresholds of 0.01 and 0.10 and was examined over a broader range of thresholds in Figure S4. Thus, the higher precision of LAWS-HiC at a common nominal threshold cannot be attributed to its empirical FDR being substantially below the nominal level.

Because differences in call-set size can themselves affect precision, we additionally compared the methods at matched numbers of calls. For each condition, the top *N* bin pairs from each method were evaluated at three values of *N*, corresponding to the number of calls made by FitHiC2, HiC-ACT, and LAWS-HiC at nominal BH-FDR 0.05 (Table S4). LAWS-HiC achieved the highest precision in all 24 matched-*N* comparisons. Because the truth-set size is fixed within each cell line-depth condition and the number of calls is fixed across methods within each matched-*N* comparison, this also corresponds to the highest recall in every case. When *N* was matched to the number of calls made by LAWS-HiC, its precision exceeded that of FitHiC2 by 0.006–0.031 and that of HiC-ACT by 0.006–0.032 across eight conditions.

Together, the fixed-threshold, empirical-FDR, and matched-call analyses show that the higher precision of LAWS-HiC cannot be explained simply by its smaller call sets or by operating at an empirical FDR substantially below the nominal level. We next examined whether called bin pairs were more enriched for biological features than not-called bin pairs within each method and whether this enrichment contrast was stronger for LAWS-HiC than for FitHiC2 and HiC-ACT.

### 3.3. LAWS-HiC’s Selective Conservativeness Is Biologically Informed

Having established that LAWS-HiC achieves higher precision than HiC-ACT and FitHiC2 at fixed FDR thresholds and matched call-set sizes, we next investigated whether the association between being called and overlap with biological features was stronger for LAWS-HiC than for the two competing methods. We addressed this through feature-overlap analysis against biological annotation data in both cell types, evaluating LAWS-HiC alongside HiC-ACT and FitHiC2 under the same criteria. The biological features tested for GM12878 included ChIA-PET CTCF interactions, ChIA-PET RNAPII interactions [25], Roadmap enhancers [28], and E-P interactions [29,30]; for mESC, the features included CTCF, H3K27ac, H3K4me1, H3K4me3 ChIP-seq peaks [31,32,33], ATAC-seq peaks [33], dbSUPER [34] and FANTOM5 [35] enhancer annotations, and E-P interactions [30,36].

**Figure 2:**
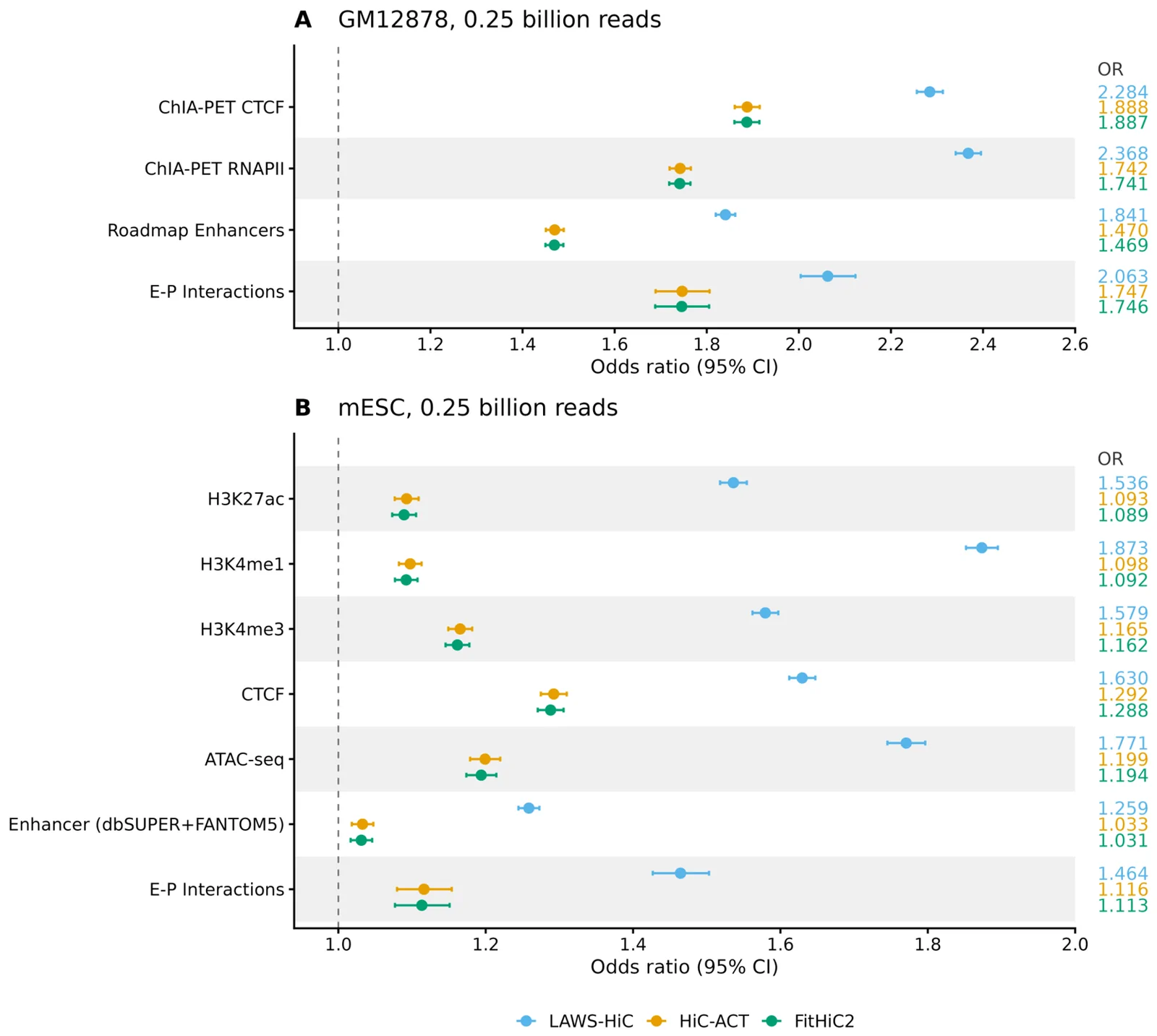
Odds ratios for biological feature overlap in called versus not-called bin pairs at 0.25 billion reads. For each method (LAWS-HiC, blue; HiC-ACT, orange; FitHiC2, green), points show the odds ratio (OR) comparing the overlap of called bin pairs with a given biological feature against the overlap of bin pairs not called by that method, and horizontal bars indicate 95% confidence intervals. The vertical dashed line marks OR = 1, corresponding to no enrichment. (**A**) GM12878. Features include ChIA-PET CTCF interactions, ChIA-PET RNAPII interactions, Roadmap enhancers (super-enhancer and typical enhancer combined), and enhancer–promoter (E-P) interactions. (**B**) mESC. Features include ChIP-seq peaks for H3K27ac, H3K4me1, H3K4me3, and CTCF; ATAC-seq peaks; enhancer regions combining dbSUPER and FANTOM5 annotations; and E-P interactions. The numerical OR estimates are shown on the right. Corresponding results for the remaining sequencing depths are shown in Figure S5, and the enhancer subcategory breakdown at 0.25 billion reads is shown in Figure S6.

For each cell line, sequencing depth, biological feature, and method, we compared feature overlap between called and not-called bin pairs using an odds ratio (OR) and its 95% confidence interval (Figure 2 and Figure S5). Counting each enhancer subcategory separately (Figure S6), this yielded 76 cell line-depth-feature comparisons per method (Table S5). For LAWS-HiC, OR was greater than 1 in all 76 comparisons, with the lower bound of the 95% confidence interval also above 1 in every case. The ORs ranged from 1.764 to 3.616 in GM12878 and from 1.255 to 1.924 in mESC. Moreover, the LAWS-HiC OR exceeded those of both HiC-ACT and FitHiC2 in all 76 comparisons. Each competing method had two comparisons for which the 95% confidence interval included 1, both involving dbSUPER enhancer–promoter interactions in mESC. At 0.25 billion reads, the OR was 1.070 for FitHiC2 and 1.073 for HiC-ACT, and at 0.50 billion reads, the corresponding ORs were 0.899 and 0.906. In the same two conditions, the ORs for LAWS-HiC were 1.604 and 1.590, respectively, with confidence intervals excluding 1.

As a complementary analysis, we also compared each method’s call set with its rejected set, defined as bin pairs called by at least one of the other two methods but not by that method (Figure S7; Table S6). This rejected set is substantially smaller than the full set of bin pairs not called by the method and focuses specifically on disagreements among the three methods. For LAWS-HiC, the OR ranged from 2.118 to 4.071 in GM12878 and from 1.328 to 2.348 in mESC, with the lower bound of the 95% confidence interval above 1 in all 76 comparisons. When the same analysis was performed for HiC-ACT and FitHiC2 using their respective rejected sets, the LAWS-HiC OR was higher in 56 of 75 evaluable comparisons relative to HiC-ACT and in 69 of 74 relative to FitHiC2. These totals are below 76 because, in one HiC-ACT comparison and two FitHiC2 comparisons, the rejected set contained no bin pairs overlapping the corresponding feature, leaving the OR undefined.

In summary, the contrast in regulatory-feature overlap between called and not-called bin pairs was consistently stronger for LAWS-HiC than for either competing method, as reflected by its higher OR in all 76 cell line-depth-feature comparisons. Thus, the smaller call set produced by LAWS-HiC at a common nominal FDR showed a stronger separation between bin pairs with and without the biological annotations examined here. Together with the higher precision reported above, these results support the use of LAWS-HiC when prioritizing a more precise and biologically enriched set of chromatin interactions is important.

We also compared the computational cost of LAWS-HiC against HiC-ACT on chromosome 10 at the shallowest sequencing depth (Table S7). Under single-core execution, the median runtime of LAWS-HiC was 434 s compared with 257 s for HiC-ACT in GM12878, and 3,049 s compared with 158 s in mESC, corresponding to 1.7- and 19.3-fold longer runtimes, respectively. The larger runtime difference in mESC is consistent with its larger TADs. Within each TAD, kernel weights for each bin pair are computed using the other bin pairs in the same TAD, so the computational cost per bin pair increases with the number of bin pairs in the TAD. The median TAD length was 1.03 Mb in mESC compared with 0.26 Mb in GM12878. LAWS-HiC parallelizes over bin pairs within each TAD, reducing the median runtime to 239 s in GM12878 and 746 s in mESC using 20 cores. Peak memory usage was comparable between LAWS-HiC and HiC-ACT: 1.15 GB and 1.82 GB, respectively, in GM12878, and 1.49 GB and 1.37 GB, respectively, in mESC. Timings are medians of three sequential repeats within a single scheduler job on an exclusively allocated node. Because the repeats shared the operating system page cache, these measurements are intended primarily for relative comparison rather than as independent runtime measurements.

## 4. Discussion

In this work, we presented LAWS-HiC, a computational tool that adapts the Locally Adaptive Weighting and Screening framework to adjust upstream Hi-C peak calling results through TAD-based spatial neighborhoods and multi-threshold sparsity estimation. LAWS-HiC consistently achieved higher PRAUC than FitHiC2 across both cell lines and all four sequencing depths; it also achieved higher PRAUC than HiC-ACT in every condition evaluated against truth peaks defined from full-depth FitHiC2 *p*-values. Because FitHiC2 also served as the upstream peak caller, however, this main truth set is not fully independent of the upstream caller. Consistent with this dependence, prevalence-rescaled PRAUC was substantially lower when performance was evaluated against orthogonal truth peaks derived from ChIA-PET and Micro-C loop calls. Nevertheless, LAWS-HiC outperformed FitHiC2 in all 20 combinations of the orthogonal truth set and sequencing depth. In contrast, its comparison with HiC-ACT varied across orthogonal truth sets, indicating that the consistent advantage over HiC-ACT observed in the main evaluation does not generalize uniformly to independent truth set definitions.

The magnitude of LAWS-HiC’s advantage over FitHiC2 varied with sequencing depth. The Hodges–Lehmann difference in per-chromosome PRAUC was larger at shallower depths and decreased overall as depth increased, from 0.016 to 0.005 for GM12878 and from 0.021 to 0.015 for mESC across the four sequencing depths. This pattern reflects how LAWS-HiC borrows information across neighboring bin pairs. At shallower depths, individual bin pairs are noisier, and the structural information carried by the local sparsity estimates within TADs contributes more. LAWS-HiC is therefore particularly advantageous for Hi-C experiments at moderate sequencing depths.

The biological enrichment analysis further demonstrates that LAWS-HiC produced a stronger contrast in regulatory-feature overlap between called and not-called bin pairs than either competing method. Across all 76 cell line-depth-feature comparisons, the odds ratio for LAWS-HiC exceeded those for both HiC-ACT and FitHiC2, indicating that its calls were more strongly enriched for the regulatory annotations examined relative to the bin pairs it did not call. For downstream analyses that benefit from biologically enriched interaction sets, such as enhancer–promoter inference or chromatin loop annotation, LAWS-HiC’s call set is therefore expected to yield a more reliable starting point than those of the competing methods.

A limitation of LAWS-HiC is its dependence on a predefined TAD partition for the spatial neighborhood. The quality of the neighborhood, and thus the local sparsity estimate, depends on the TAD calling algorithm and parameter settings used upstream. While robust TAD callers exist for both Hi-C data and other 3D contact mapping technologies, results may vary across TAD calling pipelines, and the sensitivity of LAWS-HiC to the choice of TAD partition was not systematically evaluated in this study. LAWS-HiC also requires sufficient data density for the upstream caller to perform per-bin-pair significance testing and return *p*-values. This requirement may limit its applicability to extremely sparse or degraded datasets. For example, in the PaleoHi-C library reported by Sandoval-Velasco et al. [38], chromatin loops were evaluated by aggregate peak analysis across candidate positions rather than called individually because too few contacts were recovered to resolve individual loops. In such settings, LAWS-HiC cannot be applied because the required upstream per-bin-pair *p*-values are not available, and improving loop detection at this data density would require a different analytical approach.

## 5. Conclusions

LAWS-HiC demonstrates that applying the LAWS framework to the *p*-values of an upstream Hi-C peak caller, via TAD-based neighborhoods and multi-threshold sparsity estimation, yields a more precise and biologically coherent set of chromatin interaction calls. Across two cell lines and four sequencing depths, LAWS-HiC consistently improved PRAUC over FitHiC2, with larger gains at shallower sequencing depths, and improved on HiC-ACT in all conditions evaluated against the truth set defined from full-depth FitHiC2 *p*-values. At the standard BH-FDR ≤ 0.05 threshold, LAWS-HiC showed a stronger contrast in regulatory-feature overlap between called and not-called bin pairs than either competing method across all conditions examined, supporting its utility for downstream analyses that benefit from a precise and biologically enriched set of chromatin interactions. LAWS-HiC is freely available as an R package at https://github.com/lb-zhou/LAWS_HiC (accessed on 15 September 2026).

## Figures and Tables

**Table 1:** Area under the precision–recall curve (PRAUC) of LAWS-HiC, HiC-ACT, and FitHiC2 across two cell lines and four sequencing depths. (**A**) Autosome-pooled PRAUC for each method on the raw and prevalence-rescaled scales, together with the number of candidate bin pairs and the prevalence of true interactions within the candidate set. (**B**) Per-chromosome PRAUC for each method, summarized as mean ± standard deviation (SD) across autosomes, together with paired comparisons of LAWS-HiC against FitHiC2 and HiC-ACT. Paired PRAUC differences (LAWS-HiC minus competing method) are summarized by the Hodges–Lehmann (HL) estimate and 95% confidence interval (CI); “wins” denotes the number of autosomes on which LAWS-HiC achieved a higher PRAUC than the competing method. PRAUC was computed using the PRROC R package. The truth set contains candidate bin pairs with $p<10^{-12}$ in the full-depth data, and prevalence is defined as the proportion of the candidate set belonging to the truth set. Prevalence-rescaled PRAUC is defined as (PRAUC−ρ)/(1−ρ), where ρ is the prevalence. HL estimates and 95% confidence intervals were obtained from paired per-chromosome PRAUC differences (n=22 autosomes for GM12878, n=19 for mESC).

(**A**). **Autosome-pooled PRAUC**										
		**Candidate**	**Truth Set**		**PRAUC (Raw)**			**PRAUC (Rescaled)**		
**Cell Line**	**Depth**	**Set Size**	***n***	**%**	**FitHiC2**	**HiC-ACT**	**LAWS-HiC**	**FitHiC2**	**HiC-ACT**	**LAWS-HiC**
GM12878	0.25B	765,994	622,869	81.3	0.935	0.939	0.952	0.654	0.673	0.741
	0.50B	1,171,217	830,691	70.9	0.929	0.930	0.941	0.756	0.759	0.797
	0.75B	1,328,726	882,518	66.4	0.937	0.930	0.945	0.812	0.790	0.836
	1.00B	1,422,523	905,687	63.7	0.945	0.924	0.950	0.849	0.791	0.863
mESC	0.25B	527,914	422,092	80.0	0.903	0.912	0.925	0.516	0.560	0.624
	0.50B	1,086,950	678,883	62.5	0.862	0.874	0.886	0.633	0.665	0.695
	0.75B	1,525,395	800,792	52.5	0.856	0.866	0.876	0.697	0.718	0.738
	1.00B	1,852,695	861,327	46.5	0.863	0.865	0.878	0.744	0.749	0.772
(**B**). **Per-chromosome PRAUC and paired comparisons**										
		**Per-Chromosome PRAUC (Mean ± SD)**			**LAWS-HiC vs. FitHiC2**			**LAWS-HiC vs. HiC-ACT**		
**Cell Line**	**Depth**	**FitHiC2**	**HiC-ACT**	**LAWS-HiC**	**HL**	**95% CI**	**Wins**	**HL**	**95% CI**	**Wins**
GM12878	0.25B	0.935 ± 0.009	0.939 ± 0.008	0.951 ± 0.007	0.0164	[0.0154, 0.0176]	22/22	0.0130	[0.0121, 0.0137]	22/22
	0.50B	0.930 ± 0.011	0.930 ± 0.011	0.942 ± 0.009	0.0118	[0.0106, 0.0133]	22/22	0.0114	[0.0102, 0.0122]	22/22
	0.75B	0.937 ± 0.011	0.930 ± 0.011	0.945 ± 0.009	0.0079	[0.0070, 0.0089]	22/22	0.0150	[0.0138, 0.0163]	22/22
	1.00B	0.945 ± 0.009	0.925 ± 0.011	0.951 ± 0.008	0.0052	[0.0046, 0.0058]	22/22	0.0268	[0.0252, 0.0281]	22/22
mESC	0.25B	0.902 ± 0.013	0.911 ± 0.011	0.923 ± 0.012	0.0214	[0.0200, 0.0227]	19/19	0.0124	[0.0113, 0.0137]	19/19
	0.50B	0.861 ± 0.016	0.874 ± 0.014	0.884 ± 0.015	0.0227	[0.0206, 0.0248]	19/19	0.0108	[0.0087, 0.0127]	19/19
	0.75B	0.856 ± 0.017	0.866 ± 0.016	0.875 ± 0.016	0.0188	[0.0167, 0.0211]	19/19	0.0091	[0.0065, 0.0109]	19/19
	1.00B	0.862 ± 0.016	0.865 ± 0.016	0.877 ± 0.015	0.0145	[0.0128, 0.0163]	19/19	0.0122	[0.0102, 0.0140]	19/19

**Table 2:** Precision and recall at fixed nominal BH-FDR thresholds. Precision and recall are reported for FitHiC2, HiC-ACT, and LAWS-HiC at nominal BH-FDR thresholds of 0.01, 0.05, and 0.10 across four sequencing depths in GM12878 and mESC. For each method, bin pairs with BH-adjusted *p*-values at or below the indicated threshold were treated as calls, and performance was evaluated against the truth set defined in Section 2.4.

			Precision			Recall		
Cell Line	Depth	BH-FDR	FitHiC2	HiC-ACT	LAWS-HiC	FitHiC2	HiC-ACT	LAWS-HiC
GM12878	0.25B	0.01	0.923	0.924	0.954	0.607	0.607	0.570
		0.05	0.861	0.861	0.910	0.860	0.860	0.791
		0.10	0.834	0.834	0.885	0.953	0.953	0.883
	0.50B	0.01	0.871	0.871	0.902	0.768	0.767	0.749
		0.05	0.779	0.779	0.828	0.931	0.930	0.903
		0.10	0.738	0.738	0.791	0.978	0.978	0.954
	0.75B	0.01	0.820	0.820	0.852	0.878	0.877	0.866
		0.05	0.725	0.725	0.769	0.970	0.969	0.957
		0.10	0.686	0.686	0.732	0.992	0.991	0.982
	1.00B	0.01	0.772	0.772	0.801	0.939	0.938	0.932
		0.05	0.683	0.683	0.721	0.988	0.987	0.983
		0.10	0.652	0.652	0.689	0.997	0.997	0.994
mESC	0.25B	0.01	0.884	0.885	0.959	0.564	0.562	0.363
		0.05	0.832	0.832	0.921	0.833	0.833	0.555
		0.10	0.815	0.814	0.900	0.944	0.943	0.654
	0.50B	0.01	0.796	0.796	0.900	0.683	0.681	0.537
		0.05	0.693	0.693	0.822	0.895	0.893	0.721
		0.10	0.653	0.652	0.782	0.965	0.963	0.802
	0.75B	0.01	0.731	0.731	0.839	0.777	0.774	0.668
		0.05	0.601	0.601	0.738	0.936	0.934	0.823
		0.10	0.554	0.554	0.688	0.980	0.978	0.883
	1.00B	0.01	0.676	0.677	0.782	0.845	0.843	0.767
		0.05	0.541	0.541	0.668	0.961	0.959	0.889
		0.10	0.493	0.493	0.617	0.990	0.988	0.931

## Data Availability

The original Hi-C datasets used in this study are publicly available. The GM12878 Hi-C data are available in the Gene Expression Omnibus (GEO) under accession GSE63525 [9], and the mouse embryonic stem cell (mESC) Hi-C data are available under accession GSE96107 [18]. The down-sampled contact matrices corresponding to the single random realization used at each sequencing depth in the main benchmarking analysis, which were generated without a fixed random seed, are available on Zenodo (DOI: https://doi.org/10.5281/zenodo.22308343, accessed on 15 September 2026). Scripts for down-sampling the original Hi-C data, including the scripts and fixed random seeds used to generate the five additional replicates at 0.25 billion reads in each cell line, are available in the LAWS-HiC GitHub repository at https://github.com/lb-zhou/LAWS_HiC (accessed on 15 September 2026). The biological annotation and orthogonal loop data used in this study are publicly available from their original sources. CTCF and RNAPII ChIA-PET loop data for GM12878 were obtained from Tang et al. [25]. Micro-C loop annotations for mESC called by Mustache and Chromosight were obtained from Hsieh et al. [26]. Roadmap Epigenomics enhancer annotations were obtained from the Roadmap Epigenomics Consortium [28], and GM12878 gene expression data were obtained from Schmitt et al. [29]. mESC CTCF, H3K4me1, H3K4me3, and H3K27ac ChIP-seq and ATAC-seq data were obtained from the sources described in [31,32,33]. dbSUPER super-enhancer annotations are available at https://asntech.org/dbsuper/ (accessed on 15 September 2026) [34], and FANTOM5 active enhancer annotations are available at https://fantom.gsc.riken.jp/5/ (accessed on 15 September 2026) [35]. mESC gene expression data were obtained from Li et al. [36], and transcription start sites were obtained from GENCODE [30].

## Supplementary Materials

The following supporting information can be downloaded at: https://www.mdpi.com/article/10.3390/mps9050135/s1, Supplementary Methods: detailed description of the LAWS framework [17] and its adaptation to Hi-C data, including local sparsity estimation, multi-threshold aggregation, kernel smoothing [39], treatment of small TADs, and Benjamini–Hochberg multiple-testing adjustment [21]. Supplementary Note S1: hyperparameter sensitivity analyses for the number of screening thresholds, screening-threshold endpoints, and kernel bandwidth. Supplementary Note S2: supporting analysis of precision–recall “sweet zones”. Figure S1: Gain in prevalence-rescaled PRAUC from applying LAWS-HiC to FitHiC2 and HiC-DC+ *p*-values across sequencing depths. Gain is defined as the difference in prevalence-rescaled PRAUC between LAWS-HiC and the corresponding upstream caller. Solid lines show results evaluated against the FitHiC2-based truth set, and dashed lines show results evaluated against the orthogonal truth set. The orthogonal truth set consists of the ChIA-PET union in GM12878 and Mustache Micro-C loops in mESC. Figure S2: Precision-recall curves of LAWS-HiC (blue), HiC-ACT (orange), and FitHiC2 (green) for GM12878 across all four sequencing depths. (**A**) 0.25 billion reads. (**B**) 0.50 billion reads. (**C**) 0.75 billion reads. (**D**) 1.00 billion reads. The truth set used to compute precision and recall is defined as bin pairs in the candidate set with full-depth FitHiC2 *p*-value $<10^{-12}$ (Section 2.4). Triangles mark each method’s operating point at BH-FDR ≤ 0.05. Figure S3: Precision-recall curves of LAWS-HiC (blue), HiC-ACT (orange), and FitHiC2 (green) for mESC across all four sequencing depths. (**A**) 0.25 billion reads. (**B**) 0.50 billion reads. (**C**) 0.75 billion reads. (**D**) 1.00 billion reads. The truth set definition and the interpretation of the BH-FDR ≤ 0.05 triangles are the same as in Figure S2. Figure S4: Nominal-versus-empirical FDR calibration for LAWS-HiC, HiC-ACT, and FitHiC2 across two cell lines and four sequencing depths. Empirical FDR is defined as 1−precision relative to the main-analysis truth set and is shown over nominal BH-FDR levels from 0.001 to 0.5. The dashed diagonal line represents perfect calibration, where empirical FDR equals the nominal level. Figure S5: Odds ratios for biological feature overlap in called versus not-called bin pairs across additional sequencing depths in GM12878 and mESC. Points and horizontal bars show the odds ratios (ORs) and 95% confidence intervals for LAWS-HiC, HiC-ACT, and FitHiC2, using the same feature definitions and color scheme as in Figure 2. The vertical dashed line marks OR = 1, corresponding to no enrichment. (**A**,**C**,**E**) GM12878 at 0.5, 0.75, and 1 billion reads, respectively. (**B**,**D**,**F**) mESC at the same three sequencing depths. Figure S6: Odds ratios for biological feature overlap across enhancer subcategories at 0.25 billion reads. Points and horizontal bars show the odds ratios (ORs) and 95% confidence intervals comparing called versus not-called bin pairs for LAWS-HiC, HiC-ACT, and FitHiC2, using the same color scheme as in Figure 2. The vertical dashed line marks OR = 1, corresponding to no enrichment. (**A**) GM12878. Super-enhancers (SE) and typical enhancers (TE) were obtained from the Roadmap Epigenomics Consortium; SE-P and TE-P denote enhancer-promoter (E-P) interactions in which the enhancer anchor overlaps the corresponding enhancer annotation. (**B**) mESC. Enhancer subcategories were defined using dbSUPER and FANTOM5 annotations, together with the corresponding E-P interaction subcategories. Figure S7: Comparisons of odds ratios from the call-vs-not-call and call-vs-rejected analyses. Each point is one combination of cell line, sequencing depth, and biological feature; colors indicate cell line (GM12878, purple; mESC, brown) and shapes indicate sequencing depth. Dashed lines mark the diagonal and dotted lines mark OR =1. (**A**,**B**) LAWS-HiC’s call-vs-rejected OR (*y*-axis) against FitHiC2’s (**A**) and HiC-ACT’s (**B**); points above the diagonal indicate a higher OR for LAWS-HiC. (**C–E**) For LAWS-HiC (**C**), HiC-ACT (**D**), and FitHiC2 (**E**), the call-vs-not-call OR (*y*-axis) against the call-vs-rejected OR (*x*-axis). Points for the dbSUPER E-P feature in mESC are omitted where the comparator’s OR is undefined. Figure S8: Method-specific sweet zones on the recall scale (A) and LAWS-HiC sweet zones on BH-FDR scale (B). (**A**) Sweet zones recall intervals for LAWS-HiC (blue), HiC-ACT (orange), and FitHiC2 (green) across GM12878 and mESC at four sequencing depths each; triangles mark each method’s own BH-FDR = 0.05 position, and bracketed values give each method’s own BH-FDR range within its zone. HiC-ACT’s zones are confined to BH-FDR < 0.3%; FitHiC2 has no zone in GM12878 at the two shallower depths and an extremely narrow one (width ≤ 0.004 in recall, BH-FDR $\approx 10^{-11}$–$10^{-12}$) at the two deeper depths; in mESC its zone occurs only above recall 0.99, where its own BH-FDR already exceeds 12%. (**B**) BH-FDR ranges of the LAWS-HiC sweet zones for the same eight conditions; “Universal sweet zone” is their intersection, BH-FDR ∈[0.010,0.189]. The dashed line at BH-FDR = 0.05 falls within every bar and within the universal zone. Figure S9: Precision across matched numbers of calls for LAWS-HiC, HiC-ACT, and FitHiC2. For each method, bin pairs were ranked by the method’s own *p*-values, and precision was calculated among the top *N* bin pairs over a range of matched call-set sizes. The x-axis shows *N* on a logarithmic scale. The top row shows GM12878 and the bottom row shows mESC across four sequencing depths. Vertical dashed lines indicate the number of calls made by LAWS-HiC at nominal BH-FDR ≤0.05 in each condition. Comparing methods at the same value of *N* separates differences in ranking performance from differences in the number of calls made at a common FDR threshold. Table S1: Sensitivity of PRAUC to down-sampling variability at 0.25 billion reads. (**A**) Paired differences in autosome-pooled PRAUC between LAWS-HiC and each competing method across the five replicates, including the mean, SD, minimum, and maximum. “Per-chr. wins” summarizes the number of autosomes on which LAWS-HiC achieved a higher PRAUC than the corresponding competing method, with the number in parentheses indicating the number of replicates in which LAWS-HiC won on all autosomes. (**B**) Autosome-pooled PRAUC for each method across replicates. Five independent down-sampling replicates were generated for each cell line using fixed random seeds. “Original” denotes the result from the original down-sampling realization used in the main analysis, which was generated without a fixed random seed. Random seeds were 20260801-20260805 for GM12878 and 20260811-20260815 for mESC. Table S2: Prevalence-rescaled PRAUC against orthogonal truth sets. GM12878 truth sets are CTCF and RNAPII ChIA-PET loops [25] and their union; mESC truth sets are Micro-C loops [26] called by Mustache and by Chromosight. Prevalence-rescaled PRAUC is (PRAUC−ρ)/(1−ρ), where ρ is the prevalence. Table S3: Prevalence-rescaled PRAUC with FitHiC2 and HiC-DC+ as the upstream peak callers. (**A**) Results evaluated against the truth set used in the main analysis. (**B**) Results evaluated against an orthogonal truth set, defined by the union of CTCF and RNAPII ChIA-PET loops for GM12878 and Mustache Micro-C loops for mESC. Prevalence-rescaled PRAUC is (PRAUC−ρ)/(1−ρ), where ρ is the prevalence. Table S4: Precision and recall at matched numbers of calls. For each condition the three methods are truncated to the same number of bin pairs *N*, taken as the top *N* by each method’s own *p*-values. *N* is set in turn to the number of calls each method makes at nominal BH-FDR 0.05, as indicated in the column “*N* matched to”. Table S5: Odds ratios and 95% confidence intervals for biological feature overlap in called versus non-called bin pairs. For each of the 76 cell line-depth-feature comparisons per method, the table reports the numbers of called and non-called bin pairs, the corresponding feature-overlap counts and percentages, the odds ratio (OR) and its 95% confidence interval, the confidence-interval method, and the *p*-value. The non-called set consists of all bin pairs in the candidate set not called by the corresponding method. Table S6: Odds ratios and 95% confidence intervals for biological feature overlap in called versus rejected bin pairs. For each of the 76 cell line-depth-feature comparisons per method, the table reports the numbers of called and rejected bin pairs, the corresponding feature-overlap counts and percentages, the odds ratio (OR) and its 95% confidence interval, the confidence-interval method, and the *p*-value. The rejected set consists of bin pairs not called by the corresponding method but called by at least one of the other two methods. Table S7: Runtime and memory benchmark comparing LAWS-HiC with HiC-ACT. Both methods were evaluated on chromosome 10 at 0.25 billion reads using the same exclusively allocated compute node, with three repeats per setting. Peak memory is the maximum resident set size of a single process and is therefore reported only for the single-core runs. Table S8: Comparison of standard BH applied to LAWS-adjusted *p*-values with the rejection rule from Algorithm 1 of Cai et al. [17], at nominal FDR 0.05. The Algorithm 1 rejection rule requires the local sparsity estimates $\hat{\pi }$ and is therefore applicable only to LAWS-HiC. The mean and median of $\hat{\pi }$ across the candidate set are reported for reference. Table S9: Comparison of the multi-threshold ensemble with two single-threshold rules at 0.25 billion reads. Clipping percentages are the proportion of the candidate set for which the final $\hat{\pi }$ reaches the lower ($10^{-5}$) or upper ($1-10^{-5}$) clipping bound. The empty-screening-set percentage is calculated across TADs for the single-threshold rules. Table S10: PRAUC across screening-threshold endpoint settings at 0.25 billion reads. Percentiles refer to the within-TAD *p*-value distribution. Values are means over six chromosomes, with standard deviations across chromosomes. (**A**) The lower endpoint was varied while the upper endpoint was fixed at the 95th percentile. (**B**) The upper endpoint was varied while the lower endpoint was fixed at the 20th percentile. The setting used throughout the manuscript, [20%, 95%], is shown in both panels. Table S11: PRAUC across kernel bandwidth settings at 0.25 billion reads. Raw PRAUC is reported for varying values of the bandwidth proportion α using the same six chromosomes per cell line as in the preceding analysis. Values are means across chromosomes, with standard deviations across chromosomes. The setting used throughout the manuscript, α=0.10, is included for comparison. Tables S5 and S6 are provided as a separate Excel workbook.
