# Supplementary material for: LAWS-HiC: A Locally Adaptive Weighting and Screening (LAWS) Approach to Improve Detection of Long-Range Chromatin Interactions from Hi-C Data
Source: Methods Protoc. 2026 Sep 18;9(5):135. doi: 10.3390/mps9050135 (PMC13600098; doi:10.3390/mps9050135)
Supplement: Supplementary file 1 [file mps-09-00135-s001.zip › SupplementaryMaterials-Proofread.pdf]

Supplementary Materials

Contents of Supplementary Materials

Supplementary Methods

- Locally Adaptive Weighting and Screening (LAWS) framework
- LAWS-HiC: adaptation of LAWS for Hi-C data

Supplementary Notes

- Supplementary Note S1. Hyperparameter sensitivity analyses, including the number of screening thresholds, screening-threshold endpoints, and kernel bandwidth
- Supplementary Note S2. Supporting analysis of precision-recall "sweet zones"

Supplementary Figures

- Figure S1. Gain in prevalence-rescaled PRAUC with sequencing depth using FitHiC2 and HiC-DC+ as upstream callers
- Figure S2. Precision-recall curves across four sequencing depths in GM12878
- Figure S3. Precision-recall curves across four sequencing depths in mESC
- Figure S4. Nominal-versus-empirical FDR calibration across cell lines and sequencing depths
- Figure S5. Biological feature overlap in called versus not-called bin pairs at additional sequencing depths
- Figure S6. Biological feature overlap across enhancer subcategories at 0.25 billion reads
- Figure S7. Comparison of call-vs-not-call and call-vs-rejected biological feature overlap analyses
- Figure S8. Precision-recall sweet-zone analysis
- Figure S9. Precision across matched numbers of calls for LAWS-HiC, HiC-ACT, and FitHiC2

Supplementary Tables

- Table S1. Sensitivity of PRAUC to down-sampling variability
- Table S2. Prevalence-rescaled PRAUC against orthogonal truth sets
- Table S3. Prevalence-rescaled PRAUC using FitHiC2 and HiC-DC+ as upstream callers
- Table S4. Precision and recall at matched numbers of calls
- Table S5. Biological feature overlap in called versus non-called bin pairs

- Table S6. Biological feature overlap in called versus rejected bin pairs
- Table S7. Runtime and memory benchmark
- Table S8. Sensitivity analysis using the rejection rule of Cai et al.
- Table S9. Comparison of the multi-threshold ensemble with single-threshold alternatives
- Table S10. Sensitivity to screening-threshold endpoints
- Table S11. Sensitivity to kernel bandwidth

Tables S5 and S6 are provided as a separate Excel workbook.

Supplementary Methods

Locally Adaptive Weighting and Screening (LAWS) Framework

LAWS-HiC is based on the Locally Adaptive Weighting and Screening (LAWS) framework for spatial multiple testing that incorporates local information into inference [1]. This data-driven approach consists of three steps: (1) estimating the local sparsity level, (2) constructing locally adaptive weights to adjust p-values, and (3) adjusting for multiplicity. Specifically, let $\mathbb{S}$ denote a finite, evenly spaced grid, with a hypothesis test at each location (grid cell) $s \in \mathbb{S}$. We use a binary variable $\theta(s)$ to denote the presence ($\theta(s) = 1$) or absence ($\theta(s) = 0$) of a signal of interest at location s. Define the sparsity level at location s as $\pi(s)$:

$$\pi(s) = P\{\theta(s) = 1\}. \tag{S1}$$

Let $p(s)$ be the p-value obtained from any initial hypothesis test at location s:

$$H_0(s) : \theta(s) = 0 \text{ versus } H_1(s) : \theta(s) = 1. \tag{S2}$$

To estimate the sparsity level at location s, Cai et al. [1] introduced an intermediate quantity denoted as $\pi^\tau(s)$, which approximates $\pi(s)$ given an appropriately chosen threshold τ:

$$\pi^\tau(s) = 1 - \frac{P\{p(s) > \tau\}}{1 - \tau}, \quad 0 < \tau < 1. \tag{S3}$$

Cai et al. [1] further described how to estimate $\pi^\tau(s)$ via smoothing and screening. In the smoothing step, a kernel function $K(t) : R^d \to R$ is used to pool information from neighboring locations based on their distance to s. This defines a local neighborhood weight $v_h(s, s')$:

$$v_h(s, s') = \frac{K_h(s - s')}{K_h(0)}, \quad s' \in \mathbb{S}, \tag{S4}$$

$$K_h(t) = \frac{K(t)}{h}, \tag{S5}$$

where h is the smoothing bandwidth. Next, the screening step applies the threshold τ to define a subset of likely null locations $\mathcal{T}(\tau) = \{s \in \mathbb{S} : p(s) > \tau\}$. Then the estimated sparsity level at location s, $\hat{\pi}(s)$, is further calculated as:

$$\hat{\pi}(s) = 1 - \frac{\sum_{s' \in \mathcal{T}} v_h(s, s')}{(1 - \tau) \sum_{s' \in \mathbb{S}} v_h(s, s')}. \tag{S6}$$

Once the estimated sparsity level at location s is obtained, the locally adaptive weight $\hat{w}(s)$ is constructed as:

$$\hat{w}(s) = \frac{\hat{\pi}(s)}{1 - \hat{\pi}(s)}. \tag{S7}$$

This weight is then used to calculate the adjusted p-value, $p^w(s)$:

$$p^w(s) = \min\left\{\frac{p(s)}{\hat{w}(s)},\, 1\right\}. \tag{S8}$$

LAWS-HiC: Adapting LAWS for Hi-C Data

Motivated by the LAWS framework [1], we adapt its two-dimensional formulation to Hi-C data. Let $p(ij)$ denote the initial p-value for a candidate long-range chromatin interaction between bin i and bin j, obtained from a standard Hi-C peak caller such as FitHiC2. Here i and j are bin identifiers computed as the end position of the bin (or the position of the middle point of the bin plus half of the resolution) divided by the resolution. For example, at 10 kb resolution, the genomic region 1–10,000 bp is represented by identifier $i = 1$. The LAWS-adjusted p-value is defined as

$$p^{\text{laws}}(ij) = \min\left\{\frac{p(ij)}{\hat{w}(ij)},\, 1\right\}, \tag{S9}$$

where $\hat{w}(ij)$ is the estimated locally adaptive weight at bin pair (i, j).

To construct $\hat{w}(ij)$ we first specify a neighborhood set $\mathcal{S}$ of bin pairs used for spatial pooling. Because including all bin pairs across a chromosome is computationally intractable in Hi-C, we set $\mathcal{S}$ to be all bin pairs located within the same topologically associating domain (TAD). A Gaussian kernel assigns smoothing weights

$$v(ij, mn) = \exp\left(-\frac{d^2}{2h^2}\right), \qquad d^2 = (i-m)^2 + (j-n)^2, \tag{S10}$$

where h is the smoothing bandwidth, specified below. For a threshold $\tau \in (0, 1)$ on the p-value scale, the screening subset $\mathcal{T}(\tau) = \{(i, j) \in \mathcal{S} : p(ij) > \tau\}$ collects bin pairs whose p-values are sufficiently large to be treated as candidate nulls. The local sparsity level at bin pair (i, j) is then estimated by

$$\hat{\pi}_\tau(ij) = 1 - \frac{\sum_{(m,n)\in\mathcal{T}(\tau)} v(ij, mn)}{(1-\tau)\sum_{(m,n)\in\mathcal{S}} v(ij, mn)}, \tag{S11}$$

and the locally adaptive weight is

$$\hat{w}(ij) = \frac{\hat{\pi}(ij)}{1 - \hat{\pi}(ij)}, \tag{S12}$$

where $\hat{\pi}(ij)$ is the final sparsity estimate, obtained as described below by aggregating $\hat{\pi}_\tau(ij)$ over multiple thresholds.

Motivation for Multi-Threshold Sparsity Estimation

The screening threshold τ in Equation (S11) is the tuning parameter that determines the composition of $\mathcal{T}(\tau)$. The original LAWS implementation sets τ as the Benjamini-Hochberg (BH) rejection threshold [2] applied to $\{p(ij) : (i, j) \in \mathcal{S}\}$ at a high false discovery rate (FDR) target of 0.9, with the intent that $\mathcal{T}(\tau)$ be dominated by true nulls.

However, a single threshold τ imposes a hard binary partition of $\mathcal{S}$ into a "candidate-null" subset

($p > \tau$, included in $\mathcal{T}$) and a "candidate-signal" subset ($p \leq \tau$, excluded from $\mathcal{T}$). This dichotomization is fragile in Hi-C data because the null/alternative boundary is not crisp on the p-value scale: many true chromatin interactions identified at deep sequencing depth yield only moderate p-values when the same data are analyzed at lower depth, placing them within the range occupied by null bin pairs. In the GM12878 data, for example, a bin pair with $p \approx 3 \times 10^{-49}$ at full depth (approximately 4.9 billion reads) yields $p \approx 6 \times 10^{-2}$ when the same loci pair is analyzed at 0.25 billion reads. Any single τ is therefore forced into one of two undesirable regimes: a small τ admits such moderate-signal true peaks into $\mathcal{T}(\tau)$, biasing $\hat{\pi}_\tau(ij)$ downward through contamination, whereas a large τ excludes too many bin pairs from $\mathcal{T}(\tau)$ and inflates its variance. The bias-variance trade-off formalized in the next subsection makes this concrete: no single τ can simultaneously minimize both error components. We therefore replace the single-threshold estimator with a multi-threshold ensemble that aggregates the per-threshold estimates $\hat{\pi}_{\tau_k}(ij)$ over a grid of τ values, reducing sensitivity to any single binary partition.

Bias-Variance Characterization Across Thresholds

Following Cai et al. [1], the relative bias of $\hat{\pi}_\tau(ij)$ as an estimator of $\pi(ij)$ is

$$\frac{\hat{\pi}_\tau(ij) - \pi(ij)}{\pi(ij)} = -\frac{1 - G_1(\tau \mid ij)}{1 - \tau}, \tag{S13}$$

where $G_1(\cdot \mid ij)$ is the cumulative distribution function of the p-value under the alternative at bin pair (i, j). The bias in Equation (S13) is non-positive, so $\hat{\pi}_\tau(ij)$ is a conservative underestimate of $\pi(ij)$, which is a property required for valid FDR control in the LAWS framework [1, Theorem 2 and Remark 3]. Because $G_1(\tau \mid ij) \to 0$ as $\tau \to 0$ while $G_1(\tau \mid ij) \to 1$ as $\tau \to 1$, the magnitude of the relative bias is largest at small τ and decreases monotonically as τ grows.

Conversely, the variance of $\hat{\pi}_\tau(ij)$ scales inversely with the size of the screening set:

$$\mathrm{Var}\big(\hat{\pi}_\tau(ij)\big) \;\propto\; \frac{1}{|\mathcal{T}(\tau)|}. \tag{S14}$$

Smaller τ admits more bin pairs into $\mathcal{T}(\tau)$ and yields a lower-variance estimate at the cost of greater (negative) bias from contamination by non-null bin pairs; larger τ restricts $\mathcal{T}(\tau)$ to bin pairs more likely to be true nulls, reducing bias but increasing variance. A single threshold therefore cannot simultaneously minimize both error components.

Multi-Tau Estimation via Weighted Aggregation

To exploit the bias-variance trade-off described above, we select K thresholds $\tau_1 < \tau_2 < \cdots < \tau_K$ on the p-value scale within each TAD and aggregate the resulting sparsity estimates.

Selection of the threshold grid. Within each TAD, let F_p denote the empirical distribution of the p-values $\{p(ij) : (i, j) \in \mathcal{S}\}$. We place the K thresholds at evenly spaced quantiles of F_p:

$$\tau_k = F_p^{-1}\left(\ell + \frac{k-1}{K-1}(u - \ell)\right), \quad k = 1, \ldots, K, \tag{S15}$$

with $K = 10$ thresholds, lower quantile $\ell = 0.20$, and upper quantile $u = 0.95$. Because $\mathcal{T}_k = \{(i,j) \in \mathcal{S} : p(ij) > \tau_k\}$, a threshold placed at the qth quantile retains approximately a fraction $1 - q$ of the bin pairs in the TAD. Thus, the screening sets at the lower and upper ends of the grid contain approximately 80% and 5% of the bin pairs, respectively. The two endpoints are limited by opposite considerations.

(i) Lower quantile. As shown in Equation (S13), smaller τ yields a more conservative (more negatively biased) estimate $\hat{\pi}_\tau$ [1, Section 4.1]. Because $|\mathcal{T}_k|$ decreases as τ_k increases and the aggregation weights satisfy $\omega_k \propto \sqrt{|\mathcal{T}_k|}$, the smaller thresholds also receive greater weight in the ensemble. This gives greater weight to the more conservative components, consistent with the conservative estimation condition used for FDR control in the LAWS framework [1, Theorem 2, Remark 3]. However, extending the grid too far toward lower quantiles increases the negative bias and can drive $\hat{\pi}$ to the lower clipping bound of 10^{-5} in the interval $[10^{-5}, 1-10^{-5}]$. At this bound, $\hat{w} = \hat{\pi}/(1-\hat{\pi})$ is approximately 10^{-5}, strongly downweighting the affected bin pair. This behavior is non-negligible in the sensitivity analysis: a single threshold at the midpoint of the present grid resulted in lower-bound clipping for 22.5% of the candidate set in mESC, compared with 6.0% for the $K = 10$ ensemble (Supplementary Note S1). Lowering ℓ below 0.20 would shift most of the threshold grid further toward this strongly biased regime.

(ii) Upper quantile. The upper endpoint is limited by the size of the screening set. A TAD enters the LAWS-HiC procedure only if it contains at least 20 bin pairs. At $u = 0.95$, the screening set in the smallest admissible TAD therefore contains approximately one bin pair, and increasing the upper quantile further can produce empty screening sets in small TADs. During aggregation, a threshold with an empty screening set is assigned zero weight and therefore does not produce a degenerate estimate, but it also contributes no information. The practical consequence of empty screening sets is illustrated in Supplementary Note S1, where a single-threshold rule produced empty screening sets in 10.9% of GM12878 TADs and was the only rule for which $\hat{\pi}$ reached the upper clipping bound.

For numerical handling, p-values equal to zero are replaced by the smallest representable positive double, and p-values equal to one are excluded from the empirical quantile computation.

Per-threshold sparsity estimation. For each threshold τ_k, the screening subset is $\mathcal{T}_k = \{(i,j) \in \mathcal{S} : p(ij) > \tau_k\}$, and the sparsity estimate at bin pair (i,j) is

$$\hat{\pi}_k(ij) = 1 - \frac{\sum_{(m,n)\in T_k} v(ij,mn)}{(1-\tau_k) \sum_{(m,n)\in\mathcal{S}} v(ij,mn)}. \tag{S16}$$

Each estimate $\hat{\pi}_k(ij)$ is clipped to the interval $[10^{-5}, 1-10^{-5}]$ for numerical stability.

Weighted aggregation. The final sparsity estimate is the weighted average

$$\hat{\pi}(ij) = \sum_{k=1}^{K} \omega_k \, \hat{\pi}_k(ij), \qquad \omega_k \propto \sqrt{|\mathcal{T}_k|}, \quad \sum_{k=1}^{K} \omega_k = 1. \tag{S17}$$

By Equation (S14), $\sqrt{|\mathcal{T}_k|}$ is proportional to the inverse standard error of $\hat{\pi}_k(ij)$, so the weights tilt toward more precise per-threshold estimates. We deliberately use this inverse-standard-error rule rather than the full inverse-variance rule $\omega_k \propto |\mathcal{T}_k|$ as in our setting the small-τ_k estimates have the lowest variance but the largest bias by Equation (S13), so inverse-variance weighting would over-rely on the most biased components.

Bandwidth Selection and Treatment of Small TADs

The bandwidth h controls the spatial scale over which information is pooled when estimating $\hat{\pi}(ij)$. In the original LAWS implementation, h was tuned using the cross-validation criterion `h.ccv` from the `kedd` R package [3] applied to Euclidean distances computed from the TAD midpoint. However, this criterion is designed for kernel density estimation of the distance distribution rather than for spatial smoothing of the sparsity function $\pi(ij)$, and in practice it yields bandwidths on the order of a single bin width ($h \approx 0.8$–1.3 bins at 10 kb resolution), which is insufficient to borrow information across meaningful genomic distances.

We therefore adopt a data-adaptive bandwidth proportional to the TAD span:

$$h = \max\bigl(2,\ \alpha \cdot L\bigr), \tag{S18}$$

where $L = (s_{\text{end}} - s_{\text{start}})/r$ is the TAD length in bins (with r the Hi-C resolution in base pairs) and $\alpha = 0.10$. This setting integrates information over at least 10% of the TAD's genomic span, with a minimum of 2 bins to handle small TADs. We used $\alpha = 0.10$ throughout the comparative evaluation. This value was fixed before the benchmark analysis and was not tuned against the benchmark truth set. Sensitivity to the choice of α is examined in Supplementary Note S1.

TADs shorter than 100 kb, or for which the number of bin pairs in $\mathcal{S}$ is less than 20, may contain too few bin pairs to support reliable kernel smoothing and stable multi-threshold estimation. We therefore exclude such TADs from the LAWS-HiC adjustment and retain the original FitHiC2 p-values for interactions within these TADs. Such interactions are flagged in the output and pooled together with LAWS-adjusted bin pairs in all downstream evaluation analyses.

Adjusted p-Values

Once $\hat{\pi}(ij)$ is obtained from Equation (S17), the locally adaptive weight $\hat{w}(ij)$ and the adjusted p-value $p^{\text{laws}}(ij)$ are computed via Equation (S12) and the LAWS-adjustment formula introduced above.

Supplementary Notes

Supplementary Note S1. Hyperparameter Sensitivity Analyses

Choice of the number of screening thresholds (K). LAWS-HiC estimates the local non-null proportion $\hat{\pi}$ by taking a weighted average over $K = 10$ screening thresholds placed at evenly spaced quantiles, from the 20th to the 95th percentile, of the within-TAD p-value distribution. To assess this choice, we compared the $K = 10$ ensemble with two single-threshold alternatives at 0.25 billion reads in each cell line: the midpoint of the same quantile range and the data-driven threshold rule of Cai et al. (`bh_func`) [1]. All other settings were held fixed. Because the three rules were evaluated on the same 0.25-billion-read dataset within each cell line, we used raw PRAUC for this comparison (**Table S9**).

Neither single-threshold rule performed consistently better across the two cell lines. The grid midpoint achieved the highest PRAUC in GM12878 but the lowest in mESC, whereas the Cai rule showed the opposite pattern. The $K = 10$ ensemble ranked second in both cell lines and remained close to the better-performing single-threshold rule in each case. This cross-cell-line reversal indicates that the relative performance of a single threshold can depend on the dataset, whereas the ensemble provided more consistent performance across the two datasets.

Each per-threshold sparsity estimate was clipped to $[10^{-5}, 1 - 10^{-5}]$ before aggregation, which also constrains the final weighted estimate $\hat{\pi}$ to the same interval. The ensemble also showed less frequent lower-bound clipping than the grid midpoint in both cell lines. In contrast, the Cai rule reached the upper clipping bound in GM12878, whereas no upper-bound clipping was observed for the $K = 10$ ensemble or the grid midpoint. The Cai rule can produce an empty screening set for some TADs, while the ensemble reduces the influence of an individual threshold with an empty screening set through weighted aggregation. Together, the PRAUC and clipping results support the use of the $K = 10$ ensemble as a more stable choice across datasets.

Sensitivity to screening threshold endpoints. To assess sensitivity to these choices, we varied one endpoint at a time at 0.25 billion reads while holding the other fixed. The analysis used six chromosomes from each cell line selected by stratified sampling across chromosome length: chromosomes 5, 6, 8, 10, 13, and 15 in GM12878 and chromosomes 4, 5, 8, 10, 16, and 17 in mESC. The script used for chromosome selection is available in the accompanying GitHub repository. Because all settings within each cell line used the same sequencing depth and truth set, performance was compared using raw PRAUC (Table S10).

The upper endpoint had little effect on PRAUC. Across values from the 70th to the 99th percentile, PRAUC varied by at most 0.002 in GM12878 and less than 0.001 in mESC. The lower endpoint had a larger effect, but the direction differed between cell lines: GM12878 favored a higher lower endpoint, whereas mESC favored a lower one. The default 20th-percentile lower endpoint was therefore not optimal for either dataset, but its PRAUC was within 0.005 of the best setting examined in GM12878 and less than 0.001 below the best setting in mESC. These differences were smaller than the between-chromosome variability within the default setting and smaller than the PRAUC improvement of LAWS-HiC over FitHiC2 at the same sequencing depth. Overall, the results indicate that the reported performance differences are not driven by the specific choice of threshold-grid endpoints.

Choice of the kernel bandwidth. The Gaussian kernel bandwidth was defined as a fixed proportion α of the TAD span, subject to a minimum of two bins, $h = \max(2, \alpha L)$, where L is the TAD span in bins. We used $\alpha = 0.10$ throughout the main analysis. This value was fixed before the comparative evaluation and was not tuned against the benchmark truth set.

We assessed sensitivity to α at 0.25 billion reads using the same six chromosomes per cell line as in the preceding analysis. Raw PRAUC decreased monotonically as α increased over the range examined, while the across-chromosome standard deviation increased (Table S11). Relative to the value used in the main analysis, $\alpha = 0.05$ increased PRAUC by 0.0015 in GM12878 and 0.0077 in mESC.

The difference between $\alpha = 0.05$ and $\alpha = 0.10$ is partly related to the two-bin minimum bandwidth. The floor applies to TADs shorter than $20\,\mathrm{kb}/\alpha$, corresponding to 400 kb for $\alpha = 0.05$ and 200 kb for $\alpha = 0.10$. In GM12878, 72.5% of TADs are shorter than 400 kb and 37.9% are shorter than 200 kb. Thus, at $\alpha = 0.05$, the bandwidth of most GM12878 TADs is determined by the two-bin floor rather than the proportional rule. In contrast, mESC has substantially longer TADs, with a median length of 1.03 Mb, so the floor applies less frequently.

The value $\alpha = 0.10$ is therefore close to the smallest setting at which the proportional bandwidth rule still governs most GM12878 TADs. We retained this fixed value rather than adopting the better-performing $\alpha = 0.05$ after examining the sensitivity results. HiC-ACT was likewise evaluated using the bandwidth recommended in its original publication without tuning against the benchmark truth set.

Supplementary Note S2. Supporting Analysis of Precision-Recall "Sweet Zones"

As a supporting characterization of the precision-recall curves, we defined a method-specific "sweet zone" for each combination of cell line, sequencing depth, and method. For a given method, we identified contiguous recall intervals over which its precision exceeded that of both competing methods while its BH-FDR did not exceed 0.20. The 0.20 cap was used only to prevent the search from extending into highly permissive FDR regions and does not enter LAWS-HiC or any of the primary evaluation metrics. If multiple intervals satisfied these criteria, we retained the interval with the largest integrated precision advantage over the better-performing competing method. The selected interval was defined as the representative sweet zone for that method in that condition.

Sweet-zone locations differed among the three methods across the eight cell line-depth conditions (Figure S8A). HiC-ACT had a sweet zone in all eight conditions, but these zones occurred at stringent thresholds, with its own BH-FDR remaining below 0.3%. FitHiC2 had no sweet zone in GM12878 at the two shallower depths and only very narrow zones at the two deeper depths. In mESC, its zones occurred only at recall above 0.99. In contrast, the LAWS-HiC sweet zones occurred over moderate-to-high recall ranges across all eight conditions. When expressed on the LAWS-HiC BH-FDR scale, the intersection of the eight sweet-zone ranges spanned BH-FDR 0.010–0.189 and included the nominal BH-FDR level of 0.05 used in the main analysis (Figure S8B).

Thus, at nominal BH-FDR 0.05, the LAWS-HiC operating point fell within its sweet zone in all eight conditions. Because the sweet zone is defined using relative precision dominance, however, this analysis is intended as a descriptive characterization of the precision-recall curves rather than as independent evidence of improved performance. The primary comparisons in the main text are based on PRAUC, precision at fixed FDR thresholds, matched call-set sizes, and empirical-FDR calibration.

Supplementary Figures

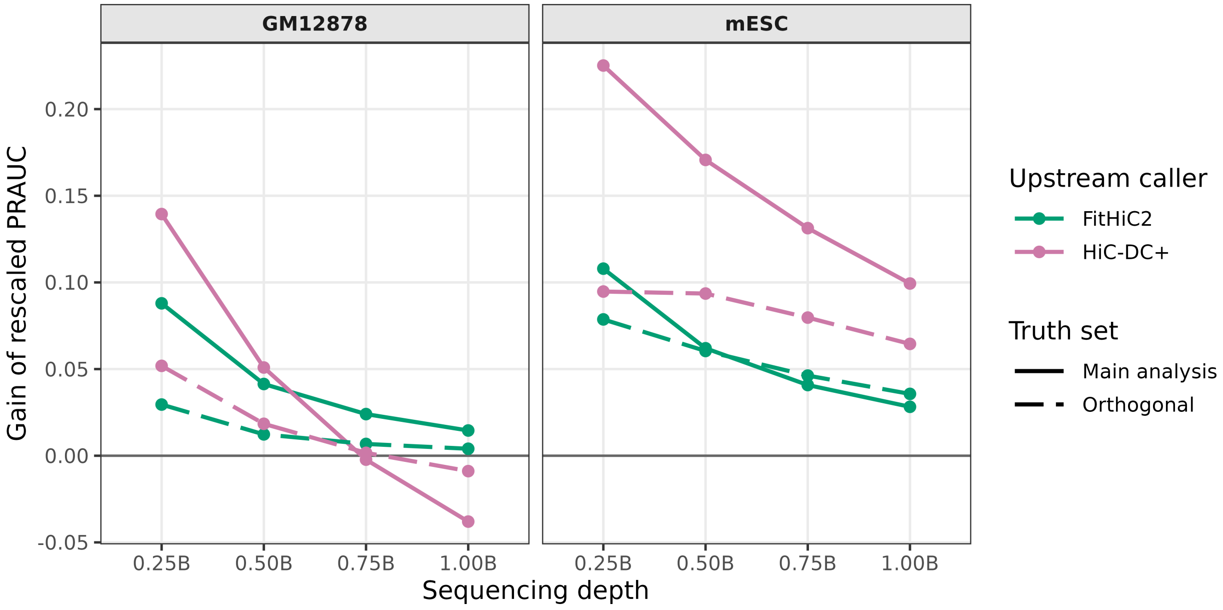

Figure S1: Gain in prevalence-rescaled PRAUC from applying LAWS-HiC to FitHiC2 and HiC-DC+ p-values across sequencing depths. Gain is defined as the difference in prevalence-rescaled PRAUC between LAWS-HiC and the corresponding upstream caller. Solid lines show results evaluated against the FitHiC2-based truth set, and dashed lines show results evaluated against the orthogonal truth set. The orthogonal truth set consists of the ChIA-PET union in GM12878 and Mustache Micro-C loops in mESC.

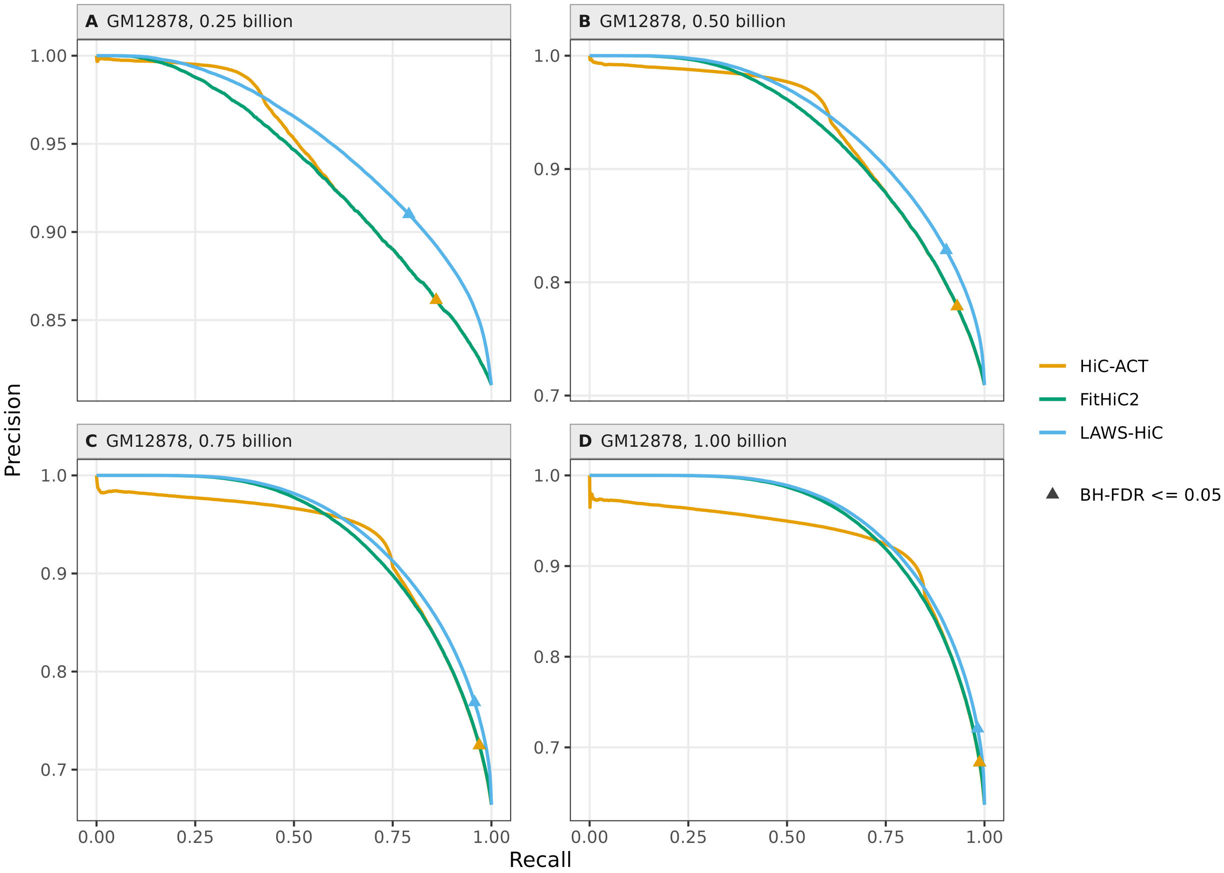

Figure S2: Precision-recall curves of LAWS-HiC (blue), HiC-ACT (orange), and FitHiC2 (green) for GM12878 across all four sequencing depths. **(A)** 0.25 billion reads. **(B)** 0.50 billion reads. **(C)** 0.75 billion reads. **(D)** 1.00 billion reads. The truth set used to compute precision and recall is defined as bin pairs in the candidate set with full-depth FitHiC2 p-value $< 10^{-12}$ (Section 2.4). Triangles mark each method's operating point at BH-FDR ≤ 0.05.

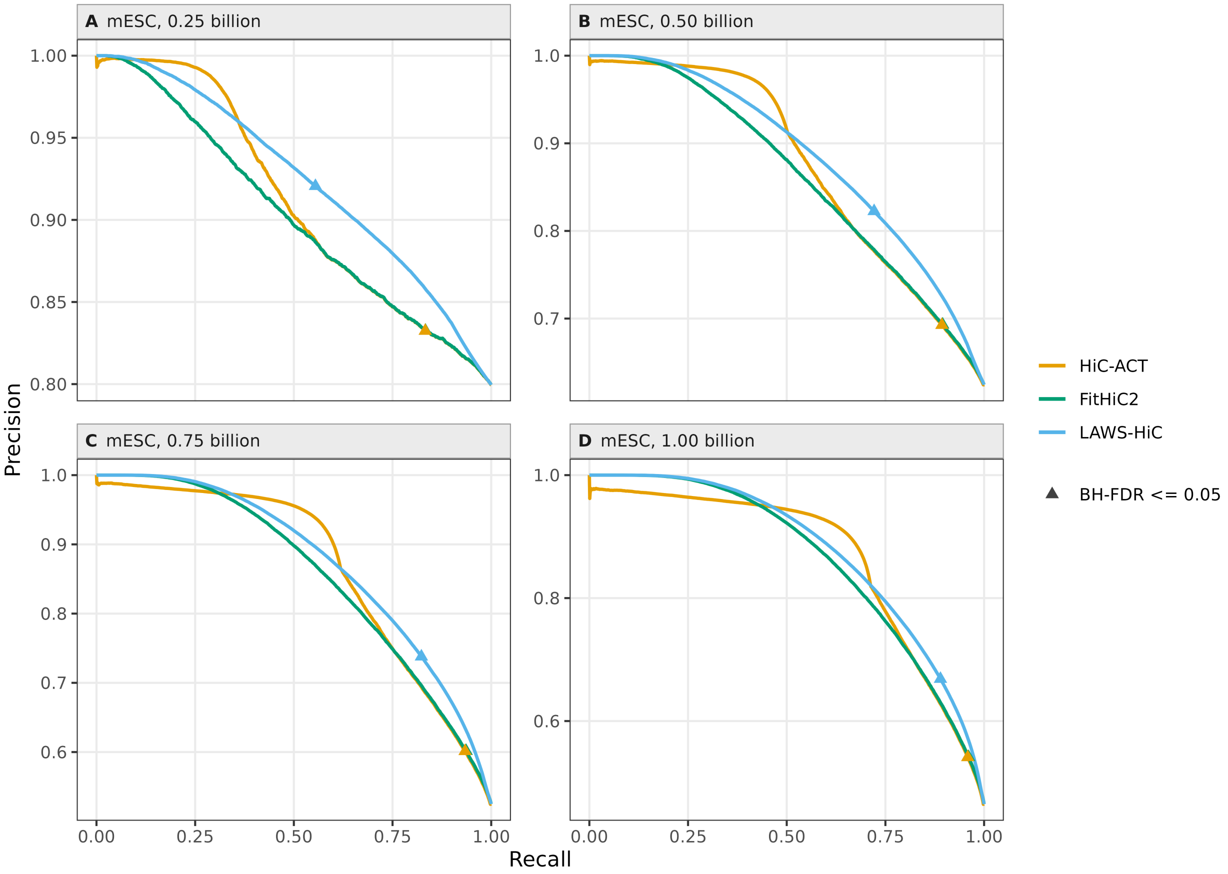

Figure S3: Precision-recall curves of LAWS-HiC (blue), HiC-ACT (orange), and FitHiC2 (green) for mESC across all four sequencing depths. **(A)** 0.25 billion reads. **(B)** 0.50 billion reads. **(C)** 0.75 billion reads. **(D)** 1.00 billion reads. The truth set definition and the interpretation of the BH-FDR ≤ 0.05 triangles are the same as in Figure S2.

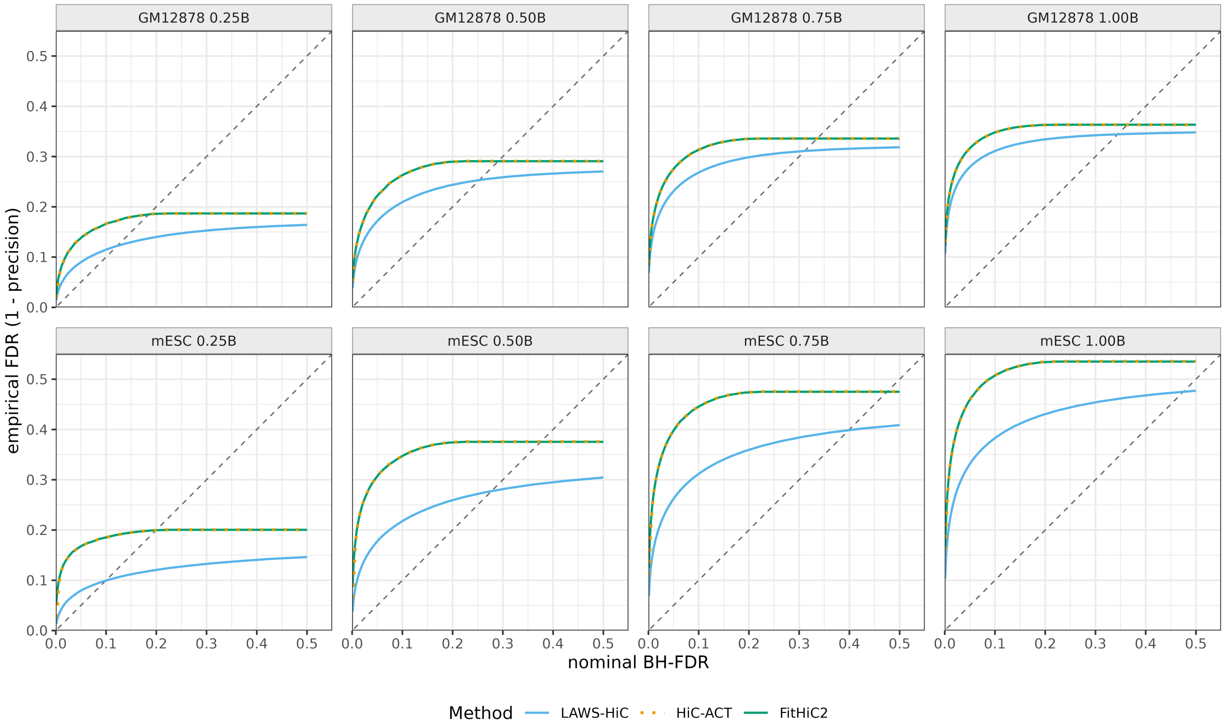

Figure S4: Nominal-versus-empirical FDR calibration for LAWS-HiC, HiC-ACT, and FitHiC2 across two cell lines and four sequencing depths. Empirical FDR is defined as $1 - \text{precision}$ relative to the main-analysis truth set and is shown over nominal BH-FDR levels from 0.001 to 0.5. The dashed diagonal line represents perfect calibration, where empirical FDR equals the nominal level.

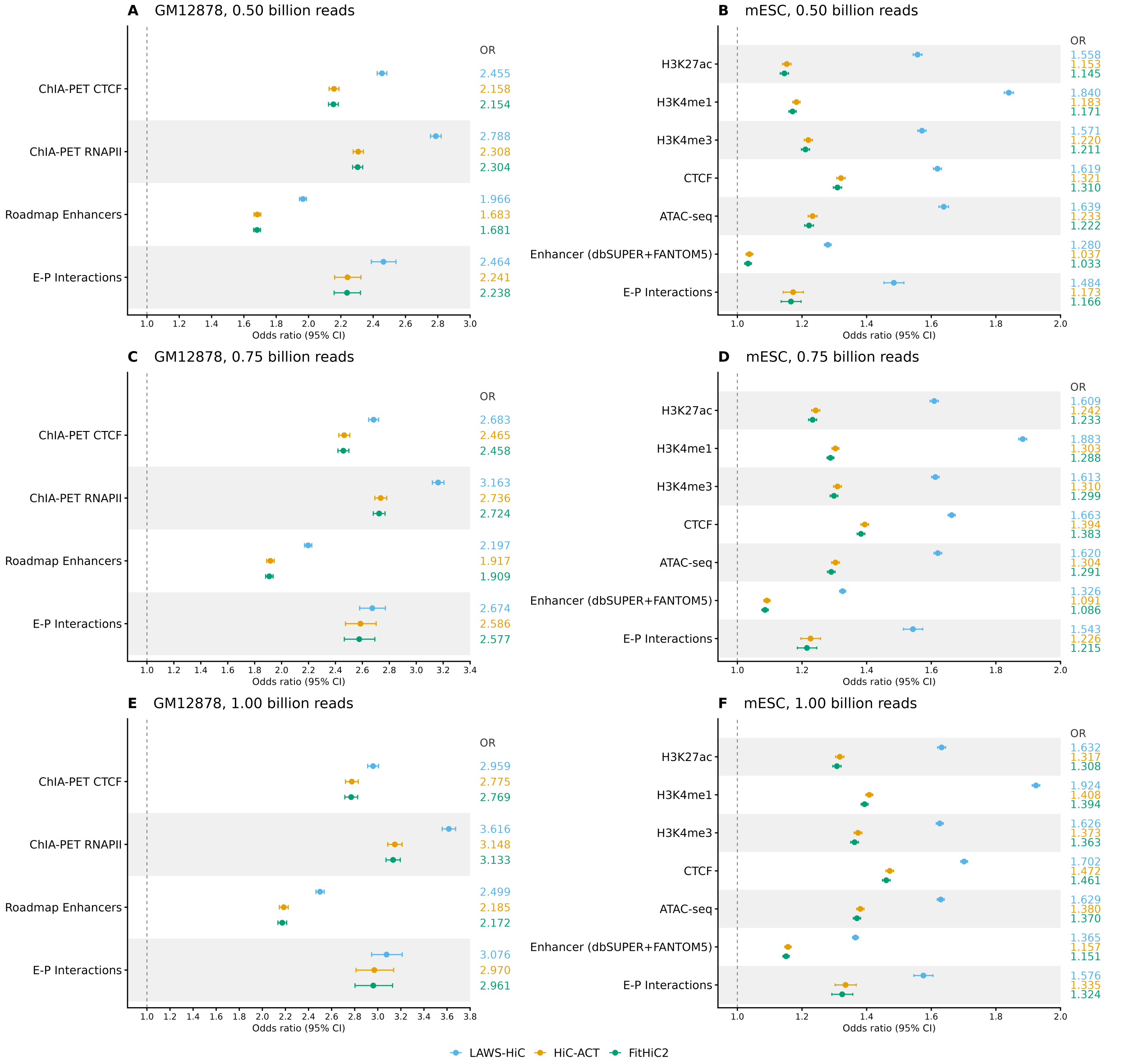

Figure S5: Odds ratios for biological feature overlap in called versus not-called bin pairs across additional sequencing depths in GM12878 and mESC. Points and horizontal bars show the odds ratios (ORs) and 95% confidence intervals for LAWS-HiC, HiC-ACT, and FitHiC2, using the same feature definitions and color scheme as in Figure 2. The vertical dashed line marks OR = 1, corresponding to no enrichment. **(A, C, E)** GM12878 at 0.5, 0.75, and 1 billion reads, respectively. **(B, D, F)** mESC at the same three sequencing depths.

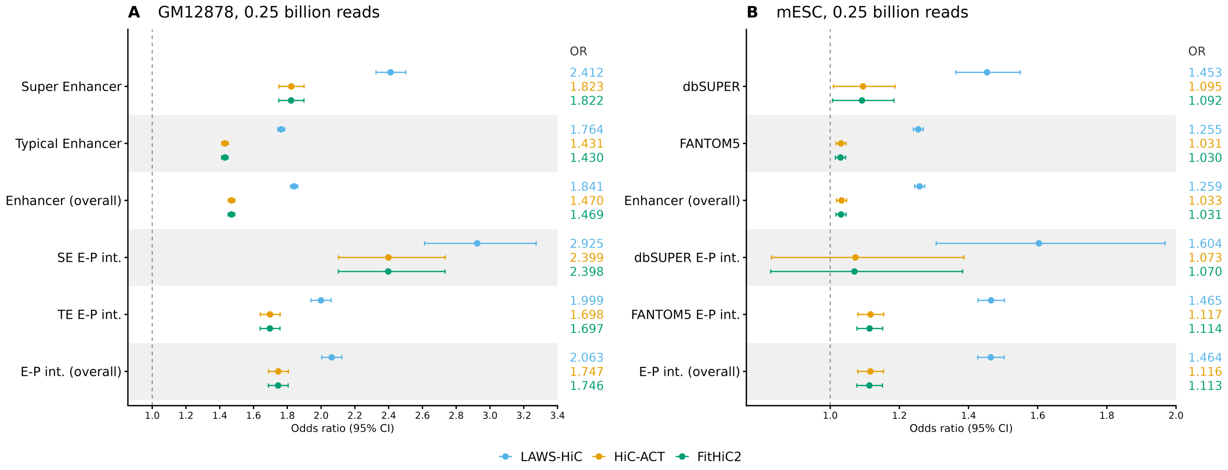

Figure S6: Odds ratios for biological feature overlap across enhancer subcategories at 0.25 billion reads. Points and horizontal bars show the odds ratios (ORs) and 95% confidence intervals comparing called versus not-called bin pairs for LAWS-HiC, HiC-ACT, and FitHiC2, using the same color scheme as in Figure 2. The vertical dashed line marks OR = 1, corresponding to no enrichment. **(A)** GM12878. Super-enhancers (SE) and typical enhancers (TE) were obtained from the Roadmap Epigenomics Consortium; SE-P and TE-P denote enhancer-promoter (E-P) interactions in which the enhancer anchor overlaps the corresponding enhancer annotation. **(B)** mESC. Enhancer subcategories were defined using dbSUPER and FANTOM5 annotations, together with the corresponding E-P interaction subcategories.

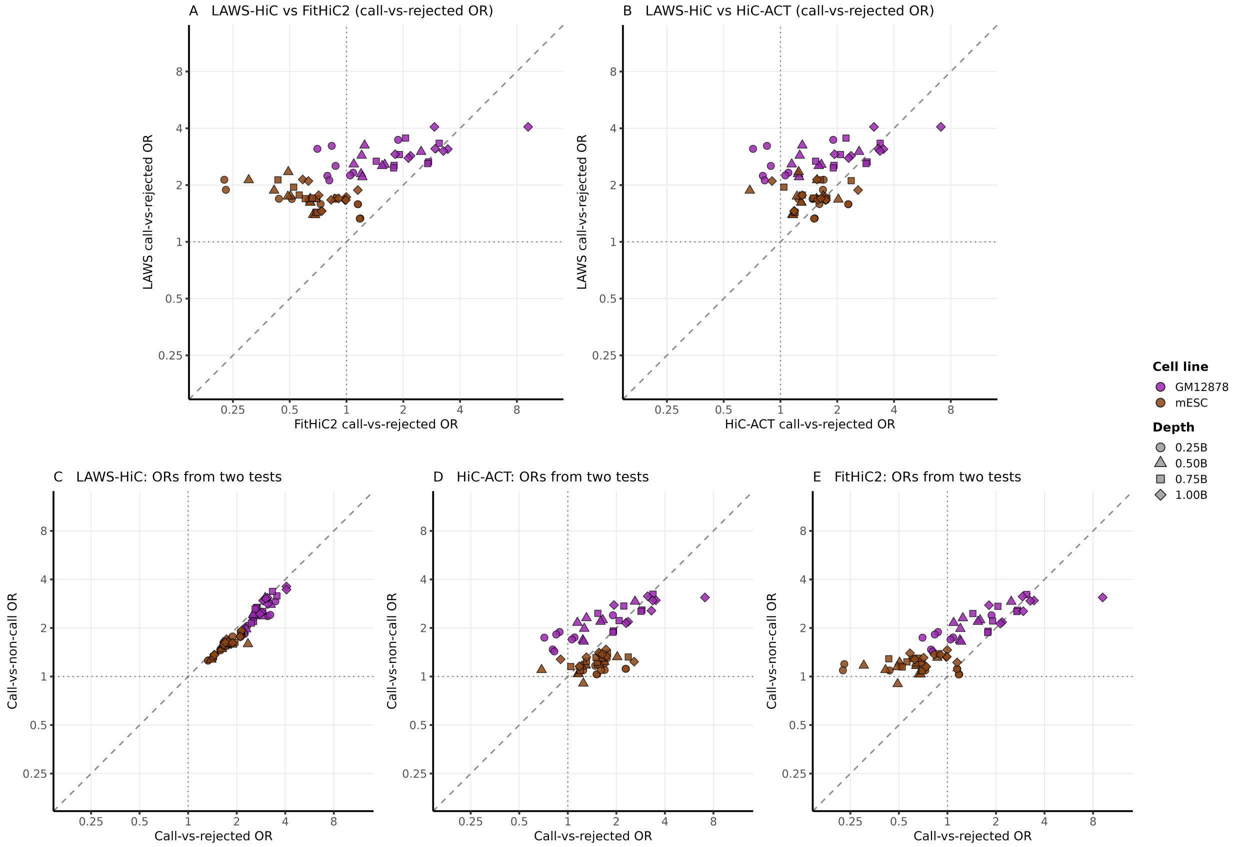

Figure S7: Comparisons of odds ratios from the call-vs-not-call and call-vs-rejected analyses. Each point is one combination of cell line, sequencing depth, and biological feature; colors indicate cell line (GM12878, purple; mESC, brown) and shapes indicate sequencing depth. Dashed lines mark the diagonal and dotted lines mark OR $= 1$. **(A, B)** LAWS-HiC's call-vs-rejected OR (y-axis) against FitHiC2's **(A)** and HiC-ACT's **(B)**; points above the diagonal indicate a higher OR for LAWS-HiC. **(C–E)** For LAWS-HiC **(C)**, HiC-ACT **(D)**, and FitHiC2 **(E)**, the call-vs-not-call OR (y-axis) against the call-vs-rejected OR (x-axis). Points for the dbSUPER E-P feature in mESC are omitted where the comparator's OR is undefined.

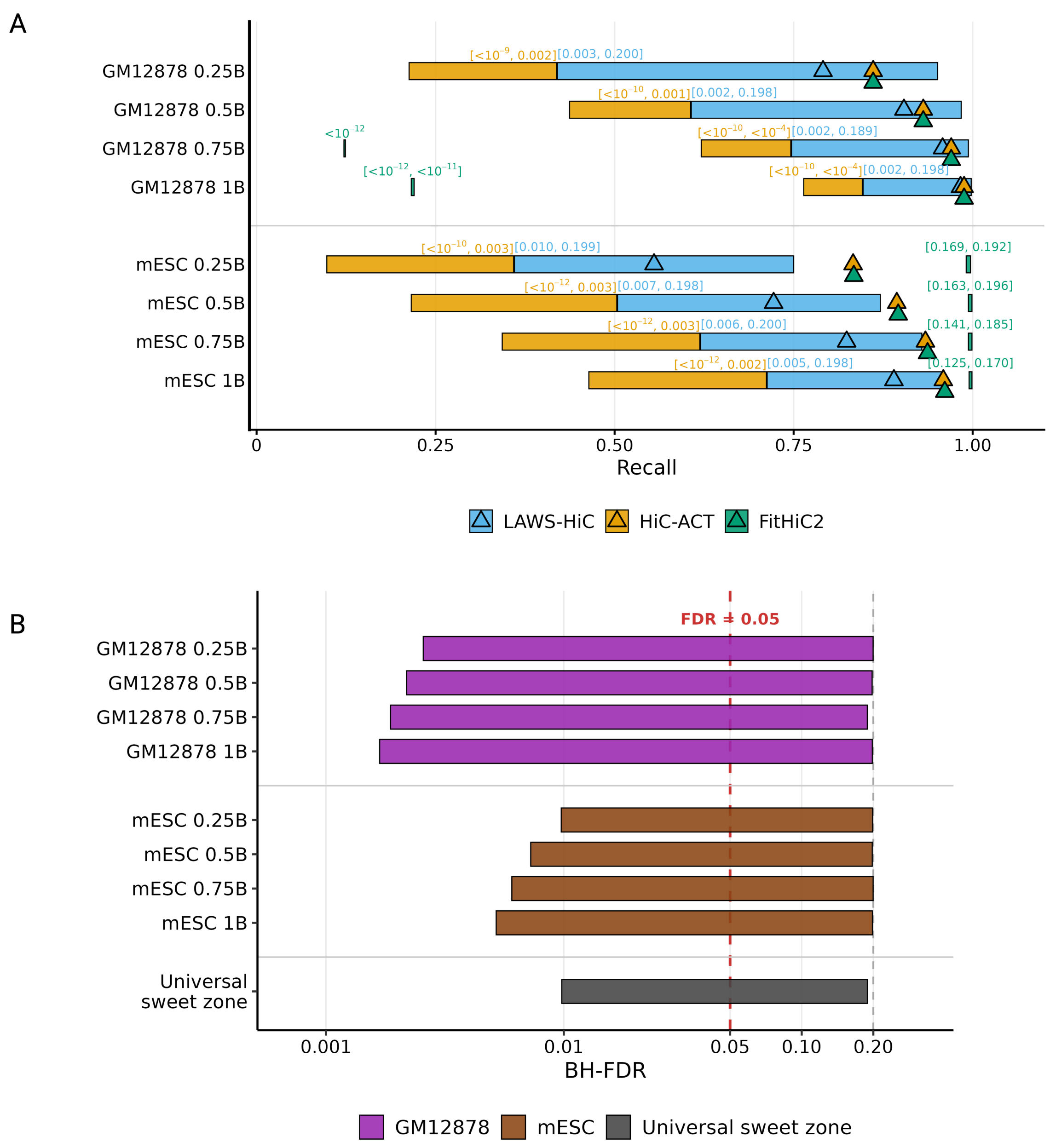

Figure S8: Method-specific sweet zones on the recall scale (A) and LAWS-HiC sweet zones on BH-FDR scale (B). **(A)** Sweet zones recall intervals for LAWS-HiC (blue), HiC-ACT (orange), and FitHiC2 (green) across GM12878 and mESC at four sequencing depths each; triangles mark each method's own BH-FDR = 0.05 position, and bracketed values give each method's own BH-FDR range within its zone. HiC-ACT's zones are confined to BH-FDR $< 0.3\%$; FitHiC2 has no zone in GM12878 at the two shallower depths and an extremely narrow one (width ≤ 0.004 in recall, BH-FDR $\approx 10^{-11}$-10^{-12}) at the two deeper depths; in mESC its zone occurs only above recall 0.99, where its own BH-FDR already exceeds 12%. **(B)** BH-FDR ranges of the LAWS-HiC sweet zones for the same eight conditions; "Universal sweet zone" is their intersection, BH-FDR $\in [0.010, 0.189]$. The dashed line at BH-FDR = 0.05 falls within every bar and within the universal zone.

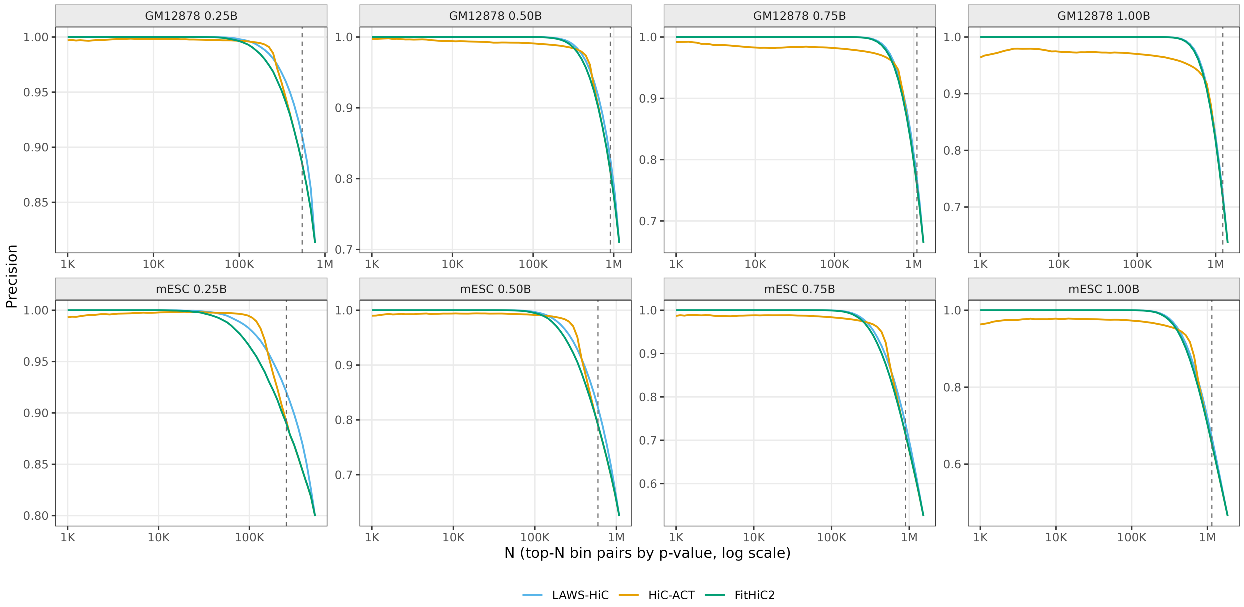

Figure S9: Precision across matched numbers of calls for LAWS-HiC, HiC-ACT, and FitHiC2. For each method, bin pairs were ranked by the method's own p-values, and precision was calculated among the top N bin pairs over a range of matched call-set sizes. The x-axis shows N on a logarithmic scale. The top row shows GM12878 and the bottom row shows mESC across four sequencing depths. Vertical dashed lines indicate the number of calls made by LAWS-HiC at nominal BH-FDR ≤ 0.05 in each condition. Comparing methods at the same value of N separates differences in ranking performance from differences in the number of calls made at a common FDR threshold.

Supplementary Tables

Table S1: Sensitivity of PRAUC to down-sampling variability at 0.25 billion reads. **(A)** Paired differences in autosome-pooled PRAUC between LAWS-HiC and each competing method across the five replicates, including the mean, SD, minimum, and maximum. "Per-chr. wins" summarizes the number of autosomes on which LAWS-HiC achieved a higher PRAUC than the corresponding competing method, with the number in parentheses indicating the number of replicates in which LAWS-HiC won on all autosomes. **(B)** Autosome-pooled PRAUC for each method across replicates. Five independent down-sampling replicates were generated for each cell line using fixed random seeds. "Original" denotes the result from the original down-sampling realization used in the main analysis, which was generated without a fixed random seed. Random seeds were 20260801-20260805 for GM12878 and 20260811-20260815 for mESC.

A. Paired differences in PRAUC

Cell line	Comparison	Mean	SD	Min	Max	Original	Per-chr. wins
GM12878	LAWS-HiC − FitHiC2	0.0164	0.000 06	0.0164	0.0165	0.0164	22/22 (5/5)
GM12878	LAWS-HiC − HiC-ACT	0.0129	0.000 08	0.0128	0.0130	0.0129	22/22 (5/5)
mESC	LAWS-HiC − FitHiC2	0.0216	0.000 23	0.0213	0.0218	0.0216	19/19 (5/5)
mESC	LAWS-HiC − HiC-ACT	0.0127	0.000 19	0.0125	0.0129	0.0128	19/19 (5/5)

B. PRAUC by method

Cell line	Method	Mean	SD	Min	Max	Original
GM12878	LAWS-HiC	0.9518	0.000 18	0.9515	0.9520	0.9517
GM12878	HiC-ACT	0.9389	0.000 13	0.9386	0.9390	0.9388
GM12878	FitHiC2	0.9353	0.000 19	0.9351	0.9355	0.9353
mESC	LAWS-HiC	0.9248	0.000 37	0.9245	0.9254	0.9246
mESC	HiC-ACT	0.9121	0.000 46	0.9117	0.9129	0.9118
mESC	FitHiC2	0.9032	0.000 50	0.9027	0.9040	0.9030

Table S2: Prevalence-rescaled PRAUC against orthogonal truth sets. GM12878 truth sets are CTCF and RNAPII ChIA-PET loops [4] and their union; mESC truth sets are Micro-C loops [5] called by Mustache and by Chromosight. Prevalence-rescaled PRAUC is $(\mathrm{PRAUC} - \rho)/(1 - \rho)$, where ρ is the prevalence.

						Prevalence-rescaled PRAUC		
Cell line	Orthogonal source	Depth	Candidate set	Truth set	Prevalence	FitHiC2	HiC-ACT	LAWS-HiC
GM12878	CTCF	0.25B	765,994	212,451	0.277	0.197	0.207	0.210
		0.50B	1,171,217	281,684	0.241	0.212	0.199	0.218
		0.75B	1,328,726	301,696	0.227	0.224	0.195	0.227
		1.00B	1,422,523	311,961	0.219	0.231	0.187	0.233
	RNAPII	0.25B	765,994	250,495	0.327	0.218	0.266	0.240
		0.50B	1,171,217	320,669	0.274	0.249	0.282	0.257
		0.75B	1,328,726	340,742	0.256	0.265	0.290	0.269
		1.00B	1,422,523	351,572	0.247	0.274	0.290	0.276
	Union	0.25B	765,994	353,678	0.462	0.288	0.328	0.317
		0.50B	1,171,217	470,436	0.402	0.321	0.338	0.333
		0.75B	1,328,726	504,623	0.380	0.342	0.344	0.349
		1.00B	1,422,523	522,627	0.367	0.354	0.342	0.358
mESC	Mustache	0.25B	527,914	268,442	0.508	0.180	0.231	0.259
		0.50B	1,086,950	503,372	0.463	0.185	0.231	0.246
		0.75B	1,525,395	650,048	0.426	0.202	0.244	0.249
		1.00B	1,852,695	744,581	0.402	0.217	0.255	0.253
	Chromosight	0.25B	527,914	263,216	0.499	0.170	0.218	0.224
		0.50B	1,086,950	472,346	0.435	0.186	0.229	0.218
		0.75B	1,525,395	600,043	0.393	0.206	0.244	0.227
		1.00B	1,852,695	682,608	0.368	0.218	0.255	0.232

Table S3: Prevalence-rescaled PRAUC with FitHiC2 and HiC-DC+ as the upstream peak callers. **(A)** Results evaluated against the truth set used in the main analysis. **(B)** Results evaluated against an orthogonal truth set, defined by the union of CTCF and RNAPII ChIA-PET loops for GM12878 and Mustache Micro-C loops for mESC. Prevalence-rescaled PRAUC is $(\mathrm{PRAUC} - \rho)/(1 - \rho)$, where ρ is the prevalence.

					FitHiC2 input		HiC-DC+ input	
Cell line	Depth	Candidate set	Truth set	Prevalence	FitHiC2	LAWS-HiC	HiC-DC+	LAWS-HiC
A. Truth set from the main analysis								
GM12878	0.25B	765,994	622,869	0.813	0.654	0.741	0.451	0.590
	0.50B	1,171,217	830,691	0.709	0.756	0.797	0.493	0.544
	0.75B	1,328,726	882,518	0.664	0.812	0.836	0.521	0.519
	1.00B	1,422,523	905,687	0.637	0.849	0.863	0.538	0.500
mESC	0.25B	527,914	422,092	0.800	0.516	0.624	0.183	0.408
	0.50B	1,086,950	678,883	0.625	0.633	0.695	0.249	0.420
	0.75B	1,525,395	800,792	0.525	0.697	0.738	0.291	0.422
	1.00B	1,852,695	861,327	0.465	0.744	0.772	0.321	0.420
B. Orthogonal truth set								
GM12878	0.25B	765,994	353,678	0.462	0.288	0.317	0.204	0.256
	0.50B	1,171,217	470,436	0.402	0.321	0.333	0.204	0.222
	0.75B	1,328,726	504,623	0.380	0.342	0.349	0.207	0.209
	1.00B	1,422,523	522,627	0.367	0.354	0.358	0.210	0.201
mESC	0.25B	527,914	268,442	0.508	0.180	0.259	0.207	0.302
	0.50B	1,086,950	503,372	0.463	0.185	0.246	0.153	0.247
	0.75B	1,525,395	650,048	0.426	0.202	0.249	0.135	0.215
	1.00B	1,852,695	744,581	0.402	0.217	0.253	0.134	0.198

Table S4: Precision and recall at matched numbers of calls. For each condition the three methods are truncated to the same number of bin pairs N, taken as the top N by each method's own p-values. N is set in turn to the number of calls each method makes at nominal BH-FDR 0.05, as indicated in the column "N matched to".

				Precision			Recall		
Cell line	Depth	N matched to	N	FitHiC2	HiC-ACT	LAWS-HiC	FitHiC2	HiC-ACT	LAWS-HiC
GM12878	0.25B	FitHiC2	622,026	0.861	0.861	0.885	0.860	0.860	0.884
		HiC-ACT	621,965	0.861	0.861	0.885	0.860	0.860	0.884
		LAWS-HiC	541,333	0.885	0.885	0.910	0.770	0.770	0.791
	0.50B	FitHiC2	992,815	0.779	0.779	0.794	0.931	0.931	0.949
		HiC-ACT	992,472	0.779	0.779	0.795	0.931	0.930	0.949
		LAWS-HiC	905,731	0.811	0.811	0.828	0.884	0.884	0.903
	0.75B	FitHiC2	1,180,648	0.725	0.724	0.733	0.970	0.969	0.981
		HiC-ACT	1,179,997	0.725	0.725	0.734	0.969	0.969	0.981
		LAWS-HiC	1,099,144	0.758	0.758	0.769	0.944	0.944	0.957
	1.00B	FitHiC2	1,309,705	0.683	0.683	0.687	0.988	0.988	0.994
		HiC-ACT	1,308,662	0.684	0.683	0.688	0.988	0.987	0.994
		LAWS-HiC	1,234,695	0.715	0.714	0.721	0.974	0.974	0.983
mESC	0.25B	FitHiC2	422,559	0.832	0.832	0.852	0.833	0.833	0.853
		HiC-ACT	422,325	0.833	0.832	0.852	0.833	0.833	0.853
		LAWS-HiC	254,328	0.890	0.893	0.921	0.536	0.538	0.555
	0.50B	FitHiC2	876,861	0.693	0.692	0.709	0.895	0.894	0.915
		HiC-ACT	875,450	0.693	0.693	0.709	0.894	0.893	0.914
		LAWS-HiC	595,421	0.791	0.790	0.822	0.694	0.693	0.721
	0.75B	FitHiC2	1,246,900	0.601	0.600	0.611	0.936	0.935	0.952
		HiC-ACT	1,243,775	0.602	0.601	0.612	0.936	0.934	0.951
		LAWS-HiC	893,321	0.715	0.714	0.738	0.798	0.796	0.823
	1.00B	FitHiC2	1,528,726	0.541	0.540	0.547	0.961	0.959	0.971
		HiC-ACT	1,525,774	0.542	0.541	0.548	0.960	0.959	0.971
		LAWS-HiC	1,146,034	0.653	0.652	0.668	0.868	0.867	0.889

Table S5. Odds ratios and 95% confidence intervals for biological feature overlap in called versus non-called bin pairs. For each of the 76 cell line-depth-feature comparisons per method, the table reports the numbers of called and non-called bin pairs, the corresponding feature-overlap counts and percentages, the odds ratio (OR) and its 95% confidence interval, the confidence-interval method, and the p-value. The non-called set consists of all bin pairs in the candidate set not called by the corresponding method.

Table S6. Odds ratios and 95% confidence intervals for biological feature overlap in called versus rejected bin pairs. For each of the 76 cell line-depth-feature comparisons per method, the table reports the numbers of called and rejected bin pairs, the corresponding feature-overlap counts and percentages, the odds ratio (OR) and its 95% confidence interval, the confidence-interval method, and the p-value. The rejected set consists of bin pairs not called by the corresponding method but called by at least one of the other two methods.

Table S7: Runtime and memory benchmark comparing LAWS-HiC with HiC-ACT. Both methods were evaluated on chromosome 10 at 0.25 billion reads using the same exclusively allocated compute node, with three repeats per setting. Peak memory is the maximum resident set size of a single process and is therefore reported only for the single-core runs.

Cell line	Method	Cores	Runtime median (s)	Range (s)	Peak memory (GB)
GM12878	HiC-ACT	1	257	257-258	1.82
	LAWS-HiC	1	434	429-438	1.15
	LAWS-HiC	20	239	232-242	-
mESC	HiC-ACT	1	158	158-159	1.37
	LAWS-HiC	1	3049	3023-3055	1.49
	LAWS-HiC	20	746	746-756	-

Table S8: Comparison of standard BH applied to LAWS-adjusted p-values with the rejection rule from Algorithm 1 of Cai et al., at nominal FDR 0.05. The Algorithm 1 rejection rule requires the local sparsity estimates $\hat{\pi}$ and is therefore applicable only to LAWS-HiC. The mean and median of $\hat{\pi}$ across the candidate set are reported for reference.

Cell line	Depth	Rule	Calls	Precision	Recall	Empirical FDR	Mean $\hat{\pi}$	Median $\hat{\pi}$
GM12878	0.25B	Standard BH	541,333	0.910	0.791	0.090	0.419	0.436
		Cai et al. rule	641,005	0.879	0.904	0.121	0.419	0.436
	0.50B	Standard BH	905,731	0.828	0.903	0.172	0.450	0.463
		Cai et al. rule	1,015,832	0.785	0.960	0.215	0.450	0.463
	0.75B	Standard BH	1,099,144	0.769	0.957	0.231	0.463	0.475
		Cai et al. rule	1,193,271	0.728	0.984	0.272	0.463	0.475
	1.00B	Standard BH	1,234,695	0.721	0.983	0.279	0.471	0.483
		Cai et al. rule	1,311,651	0.686	0.994	0.314	0.471	0.483
mESC	0.25B	Standard BH	254,328	0.921	0.555	0.079	0.218	0.190
		Cai et al. rule	370,682	0.875	0.769	0.125	0.218	0.190
	0.50B	Standard BH	595,421	0.822	0.721	0.178	0.261	0.240
		Cai et al. rule	793,363	0.743	0.868	0.257	0.261	0.240
	0.75B	Standard BH	893,321	0.738	0.823	0.262	0.281	0.264
		Cai et al. rule	1,140,028	0.648	0.922	0.352	0.281	0.264
	1.00B	Standard BH	1,146,034	0.668	0.889	0.332	0.296	0.282
		Cai et al. rule	1,416,640	0.580	0.954	0.420	0.296	0.282

Table S9: Comparison of the multi-threshold ensemble with two single-threshold rules at 0.25 billion reads. Clipping percentages are the proportion of the candidate set for which the final $\hat{\pi}$ reaches the lower (10^{-5}) or upper ($1 - 10^{-5}$) clipping bound. The empty-screening-set percentage is calculated across TADs for the single-threshold rules.

Cell line	Rule	PRAUC	Clipped low (%)	Clipped high (%)	Empty screening set (%)
GM12878	$K = 10$ grid	0.952	0.99	0.00	–
	grid midpoint	0.952	3.43	0.00	0.00
	Cai rule	0.948	6.87	1.81	10.93
mESC	$K = 10$ grid	0.925	5.99	0.00	–
	grid midpoint	0.921	22.54	0.00	0.00
	Cai rule	0.926	5.01	0.00	2.69

Table S10: PRAUC across screening-threshold endpoint settings at 0.25 billion reads. Percentiles refer to the within-TAD p-value distribution. Values are means over six chromosomes, with standard deviations across chromosomes. **(A)** The lower endpoint was varied while the upper endpoint was fixed at the 95th percentile. **(B)** The upper endpoint was varied while the lower endpoint was fixed at the 20th percentile. The setting used throughout the manuscript, [20%, 95%], is shown in both panels.

Grid endpoints	GM12878	mESC
A. Lower endpoint varied		
$[90\%, 95\%]$	0.9556 ± 0.0042	0.9149 ± 0.0163
$[80\%, 95\%]$	0.9557 ± 0.0045	0.9197 ± 0.0156
$[70\%, 95\%]$	0.9551 ± 0.0048	0.9225 ± 0.0148
$[60\%, 95\%]$	0.9543 ± 0.0051	0.9243 ± 0.0140
$[50\%, 95\%]$	0.9534 ± 0.0052	0.9255 ± 0.0135
$[40\%, 95\%]$	0.9525 ± 0.0054	0.9266 ± 0.0131
$[30\%, 95\%]$	0.9516 ± 0.0054	0.9278 ± 0.0129
$[25\%, 95\%]$	0.9511 ± 0.0055	0.9285 ± 0.0127
$[20\%, 95\%]$	0.9507 ± 0.0055	0.9290 ± 0.0124
$[15\%, 95\%]$	0.9503 ± 0.0056	0.9292 ± 0.0122
$[10\%, 95\%]$	0.9499 ± 0.0056	0.9294 ± 0.0120
$[5\%, 95\%]$	0.9496 ± 0.0057	0.9294 ± 0.0121
B. Upper endpoint varied		
$[20\%, 99\%]$	0.9509 ± 0.0055	0.9289 ± 0.0124
$[20\%, 95\%]$	0.9507 ± 0.0055	0.9290 ± 0.0124
$[20\%, 90\%]$	0.9505 ± 0.0055	0.9289 ± 0.0124
$[20\%, 85\%]$	0.9502 ± 0.0056	0.9288 ± 0.0124
$[20\%, 80\%]$	0.9499 ± 0.0056	0.9287 ± 0.0123
$[20\%, 70\%]$	0.9492 ± 0.0056	0.9284 ± 0.0123

Table S11: PRAUC across kernel bandwidth settings at 0.25 billion reads. Raw PRAUC is reported for varying values of the bandwidth proportion α using the same six chromosomes per cell line as in the preceding analysis. Values are means across chromosomes, with standard deviations across chromosomes. The setting used throughout the manuscript, $\alpha = 0.10$, is included for comparison.

α	GM12878	mESC
0.05	0.9522 ± 0.0054	0.9367 ± 0.0109
0.10	0.9507 ± 0.0055	0.9290 ± 0.0124
0.15	0.9493 ± 0.0057	0.9240 ± 0.0136
0.20	0.9483 ± 0.0059	0.9204 ± 0.0142
0.30	0.9471 ± 0.0060	0.9160 ± 0.0151
0.50	0.9463 ± 0.0061	0.9126 ± 0.0156

References

[1] Cai, T.T.; Sun, W.; Xia, Y. LAWS: A locally adaptive weighting and screening approach to spatial multiple testing. *J. Am. Stat. Assoc.* **2022**, *117*, 1370–1383.

[2] Benjamini, Y.; Hochberg, Y. Controlling the false discovery rate: a practical and powerful approach to multiple testing. *J. R. Stat. Soc. Ser. B Methodol.* **1995**, *57*, 289–300.

[3] Guidoum, A.C. Kernel Estimator and Bandwidth Selection for Density and Its Derivatives: The kedd Package, 2020, [2012.06102]. arXiv:2012.06102.

[4] Tang, Z.; Luo, O.J.; Li, X.; Zheng, M.; Zhu, J.J.; Szalaj, P.; Trzaskoma, P.; Magalska, A.; Wlodarczyk, J.; Ruszczycki, B.; et al. CTCF-Mediated Human 3D Genome Architecture Reveals Chromatin Topology for Transcription. *Cell* **2015**, *163*, 1611–1627.

[5] Hsieh, T.H.S.; Cattoglio, C.; Slobodyanyuk, E.; Hansen, A.S.; Darzacq, X.; Tjian, R. Enhancer-promoter interactions and transcription are largely maintained upon acute loss of CTCF, cohesin, WAPL or YY1. *Nature genetics* **2022**, *54*, 1919–1932.
